# Quantitative trait loci analysis of glucosinolate, sugar, and organic acid concentrations in *Eruca vesicaria* subsp. *sativa*

**DOI:** 10.1186/s43897-022-00044-x

**Published:** 2022-10-10

**Authors:** Luke Bell, Martin Chadwick, Manik Puranik, Richard Tudor, Lisa Methven, Carol Wagstaff

**Affiliations:** 1 School of Agriculture, Policy & Development, Crop Sciences, University of Reading, Reading, UK; 2 School of Chemistry, Food & Pharmacy, Food & Nutritional Sciences, University of Reading, Reading, UK; 3 Elsoms Seeds Ltd., Spalding, UK

**Keywords:** Rocket, Arugula, Brassicaceae, Glucoraphanin, Malic acid, 4-methoxyglucobrassicin

## Abstract

*Eruca vesicaria* subsp. *sativa* is a leafy vegetable of the Brassicaceae family known for its pungency. Variation in growing conditions, leaf age, agronomic practices, and variety choice lead to inconsistent quality, especially in content of isothiocyanates (ITCs) and their precursor glucosinolates (GSLs). We present the first linkage and Quantitative Trait Loci (QTL) map for *Eruca*, generated using a population of 139 F_4_ lines. A significant environmental effect on the abundance of primary and secondary metabolites was observed, with UK-grown plants containing significantly higher concentrations of glucoraphanin, malic acid, and total sugars. Italian-grown plants were characterized by higher concentrations of glucoerucin, indolic GSLs, and low monosaccharides. 20 QTL were identified and associated with robust SNP markers. Five genes putatively associated with the synthesis of the GSL 4-methoxyglucobrassicin (4MGB) were identified as candidate regulators underlying QTL. Analysis revealed that orthologs of *MYB51*, *IGMT1* and *IGMT4* present on LG1 are associated with 4MGB concentrations in *Eruca*. This research illustrates the utility of the map for identifying genes associated with nutritional composition in *Eruca* and its value as a genetic resource to assist breeding programs for this leafy vegetable crop.

**Supplementary Information:**

The online version contains supplementary material available at 10.1186/s43897-022-00044-x.

## Core

The first linkage map for *Eruca* is presented with phytochemical characterization of 139 mapping population lines across two growing locations. Plants grown in the UK and Italy are phytochemically distinct, with UK plants accumulating significantly more monosaccharides, malic acid, and glucoraphanin, whereas Italy grown plants contained significantly more glucosativin and sucrose. Robust markers associated with these phytochemical traits have been identified. Genes associated with transcriptional regulation and biosynthesis of 4-methoxyglucobrassicin have been identified underlying QTL.

## Gene & Accession Numbers

Genome sequence data studied in this article can be found at the European Nucleotide Archive under accession number GCA_932364175. A previously reported copy of *MYB51* (NCBI accession number JX946185) was mapped.

## Introduction

*Eruca vesicaria* subsp. *sativa* (known as ‘salad’ rocket or arugula) is a leafy vegetable of the Brassicaceae family and is notable for its pungent flavor. It is an annual plant (Hanin et al. 2013) closely related to *Arabidopsis thaliana* and *Brassica oleracea* (Bell and Wagstaff 2017). It is cultivated commercially and by amateur growers across the world, and has potential for development as a health beneficial crop that is tolerant to a range of environmental conditions (Jasper et al. 2020; Westberg et al. 2013). Quality and nutritional traits of rocket are inconsistent, compromising consumer acceptability of the crop. Breeders aim to improve the environmental stability of phytochemical traits underlying these attributes, but the genetic loci have not been previously identified. The species has comparable genome size to *B. oleracea* and *Raphanus sativus* (Bell et al. 2020a) and has many biochemical similarities to the genetically distinct ‘wild’ rocket, *Diplotaxis* genus. The *Eruca* genome may therefore prove to be useful in understanding orthologous genetic mechanisms underlying nutritional and organoleptic traits in these related leafy vegetable species. *Eruca* has been proposed as a promising genetic resource for future development (Pignone and Gómez-Campo 2011).

**Fig. 1:**
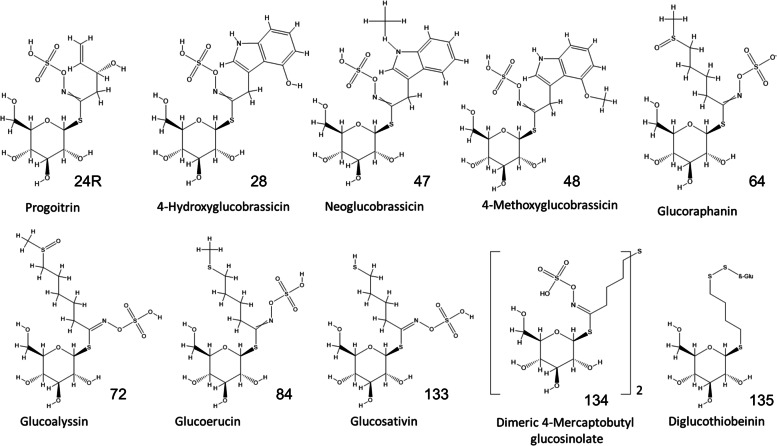
Chemical structure of glucosinolate compounds found in *Eruca vesicaria* subsp. *sativa* leaves. Inset numbers refer to those assigned by Blažević et al. (2019)

Taste and flavor of ‘salad’ rocket is complex and influenced by crop and consumer genetics, abiotic and biotic stresses, season, and cultivation practices (Bell et al. 2020b). Two components determining sensory attributes and quality are glucosinolates (GSLs) and sugars. GSLs are a diverse group of molecules found in the order Brassicales based on a *S*-*β-*_D_-glucopyrano unit anomerically bound to an *O*-sulfated (*Z*)-thiohydroximate group (Blažević et al. 2019). ‘Salad’ rocket contains a high number of different GSLs (23 have been reported; Bell et al. 2021), made up predominantly of aliphatic compounds such as glucoraphanin (GRA; 4-(methylsulfinyl)-butyl GSL), glucoerucin (GER; 4-(methylthio)-butyl GSL), and glucosativin (GSV; 4-mercaptobutyl GSL; Fig. 1). GSV is unusual in that it is present within leaves as a dimer (dimeric 4-mercaptobutyl GSL; DMB) and a monomer. Rocket also contains other unusual GSL compounds such as glucorucolamine (GRM; 4-(cystein-*S*-yl)-butyl GSL) and diglucothiobeinin (DGTB; 4-(β-_D_-glucopyranosyldisulfanyl)-butyl GSL) (Bell et al. 2021). The genes responsible for the synthesis of GSV, DMB, GRM and DGTB are unknown. Rocket leaves also contain indolic GSLs such as glucobrassicin (GBC; indolyl-3-methyl GSL), 4-methoxyglucobrassicin (4MGB; 4-methoxyindolyl-3-methyl GSL), and neoglucobrassicin (NGB; 1-methoxyindolyl-3-methyl GSL). These typically occur in lower concentrations than aliphatic GSLs in *Eruca*, but the pathway of their synthesis is more fully elucidated. Several of these compounds are present in model plants *A. thaliana* and *B. oleracea* (GRA, GER, GBC, 4MGB, and NGB; Fahey et al. 2001) and the genetic resources presented in this study represent an opportunity for enhanced understanding of their metabolism and control in *Eruca*.

Previous studies have found that the ratio between GSLs, their hydrolysis products (GHPs), and sugars is an important determinant in the taste and acceptance of rocket leaves (Bell et al. 2017). The relative abundance of sugars is associated with sweeter, milder tastes, which are preferred by the majority of consumers (Bell et al. 2020b). Consideration of cultivars and their sugar profiles is therefore important for the purposes of breeding for improved taste and flavor.

Organic acids are a metabolic component that remain largely unstudied Brassicaceae. Citric, malic, and succinic acids are involved in primary metabolism as part of the tricarboxylic acid (TCA) cycle and the generation of metabolic energy (Ludwig 2016). The TCA cycle also produces precursors to amino acids like methionine, which is an important component of aliphatic GSL biosynthesis (Malitsky et al. 2008). Like *A. thaliana*, rocket is known to accumulate high organic acid concentrations (Beale and Sussman 2018; Bell et al. 2017) and increased abundances within leaves may be an indicator of metabolic stress (Shi et al. 2015).

Greater accessibility of full genome sequencing has provided the opportunity for making physical genetic maps of ‘niche’ species. However, linkage maps remain of practical utility to researchers and plant breeders to facilitate linkage between traits of interest and genetic markers, with the most powerful type of genetic marker being located within a gene that regulates the variation observed in the trait. Establishing linkage leads to more relevant marker selection when gene function is unknown, or the gene driving variation in the mapping population is unknown (such as for GSV concentrations). To date no linkage or Quantitative Trait Loci (QTL) maps have been produced in *Eruca*. QTL mapping is used for the dissection of complex traits that may be influenced by environmental factors (Jones et al. 1997). The approach associates phenotypic variance with genetic markers, such as single nucleotide polymorphisms (SNPs). To breed health beneficial and better tasting rocket cultivars it is essential to develop genetic markers associated with the synthesis of relevant compounds and genetic pathways.

In the present work we report a genetic linkage and QTL map for GSL, sugar, and organic acid concentrations within *Eruca* leaves. The aims of the study were: to determine the impacts of cultivation environment upon metabolite abundances in a segregating population of plants, and to identify QTL and underlying genes that may be utilized in breeding ‘salad’ rocket for improved quality traits. A mapping population of 139 lines was produced from parent lines that segregated for the traits of interest, and was grown in the United Kingdom and Italy to test the following hypotheses: 1) Plants grown in Italy contain higher concentrations of GSLs, based on previous evidence showing greater accumulations in high temperature environments (Jasper et al. 2020). 2) Plants grown in the UK contain greater concentrations of sugars as a result of lower ambient temperatures initiating abiotic stress responses (Steindal et al. 2015). 3) Plants grown in the UK contain higher organic acid concentrations compared to Italy-grown plants due to the less favorable climate (lower temperature and higher humidity) and increased oxidative stress (Igamberdiev and Eprintsev 2016). 4) Genetic loci associated with GSLs, sugars, and organic acids, and underlying candidate genes can be identified and used as molecular markers for future breeding efforts.

## Results

### Glucosinolate, sugar, and organic acid compositions

**Fig. 2:**
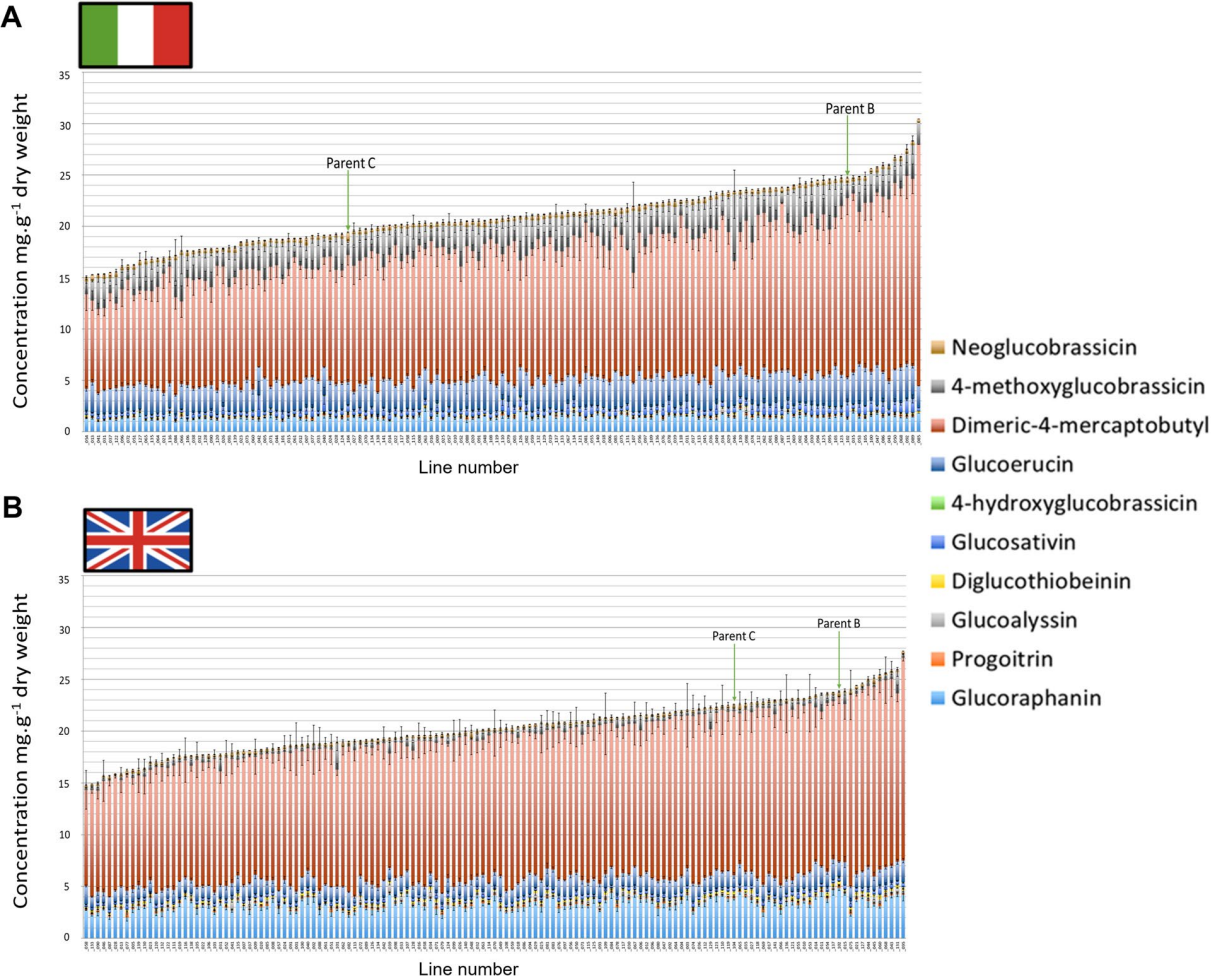
Glucosinolate compositions and concentrations (mg g^− 1^ dry weight) of 139 F_4_ *Eruca vesicaria* subsp. *sativa* mapping population lines and their parental lines B and C cultivated in Italy (**A**) and the United Kingdom (**B**). Lines are ordered along the x-axis according to total glucosinolate concentration

**Fig. 3:**
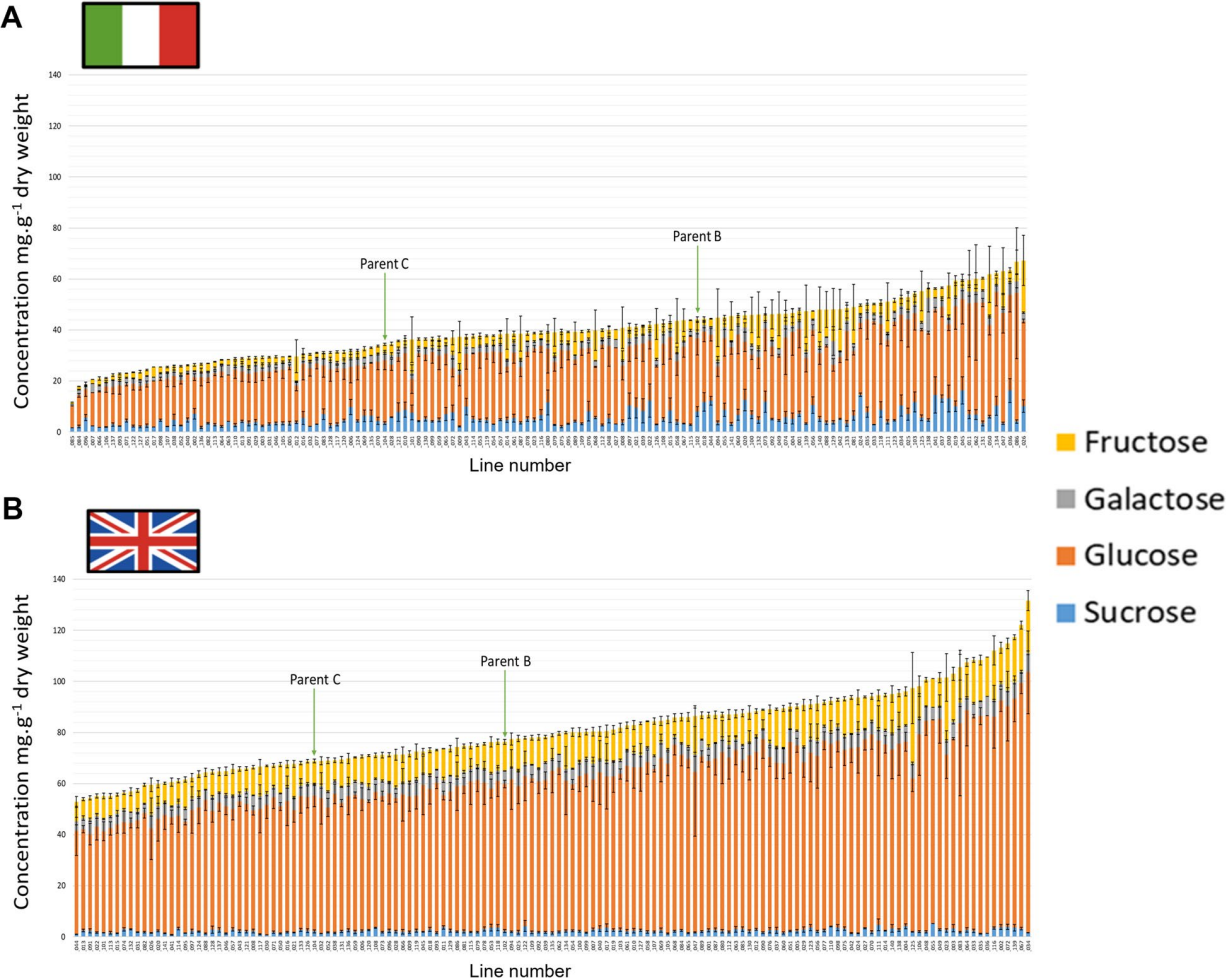
Sugar compositions and concentrations (mg g^− 1^ dry weight) of 139 F_4_ *Eruca vesicaria* subsp. *sativa* mapping population lines and their parental lines B and C cultivated in Italy (**A**) and the United Kingdom (**B**). Lines are ordered along the x-axis according to total sugar concentration

**Fig. 4:**
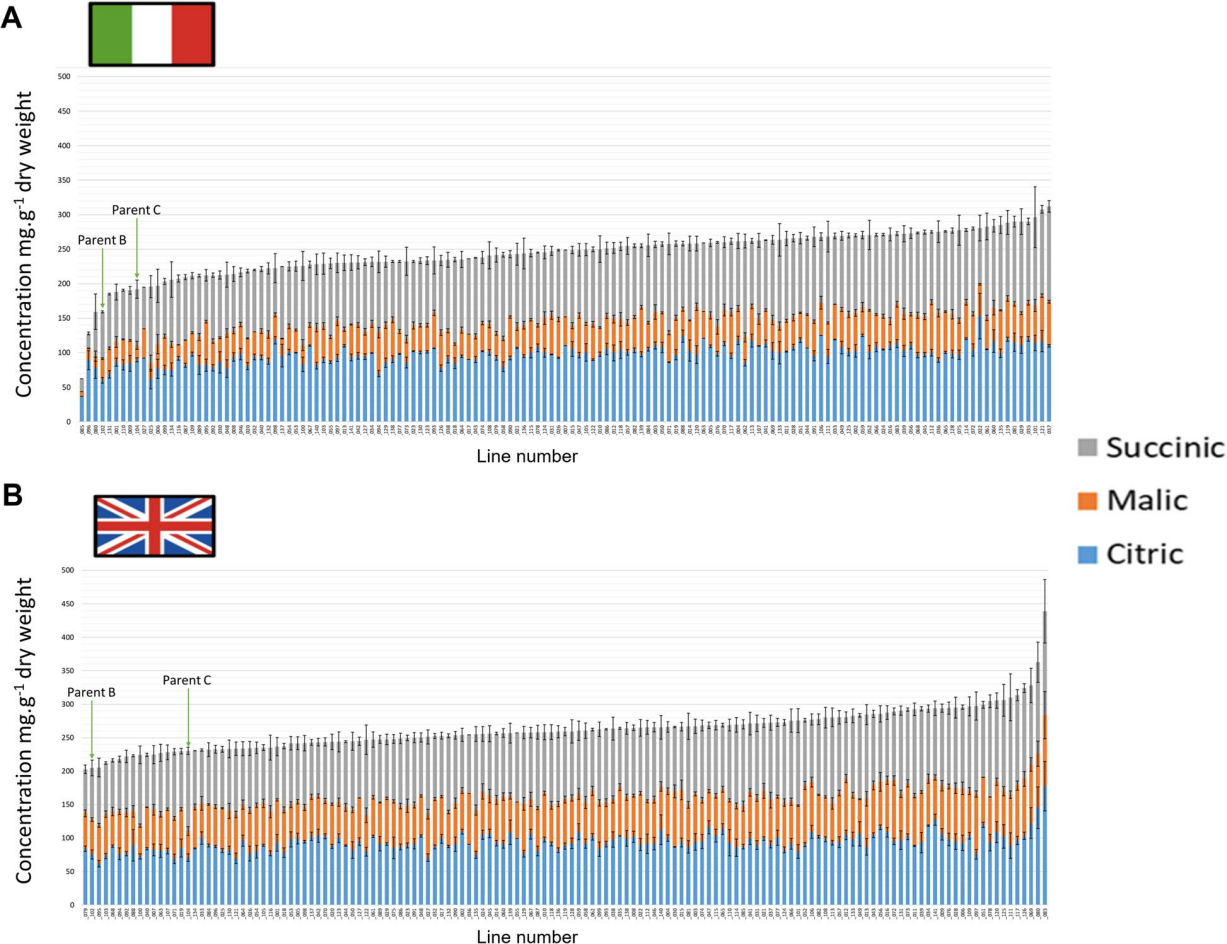
Organic acid compositions and concentrations (mg g^− 1^ dry weight) of 139 F_4_ *Eruca vesicaria* subsp. *sativa* mapping population lines and their parental lines B and C cultivated in Italy (**A**) and the United Kingdom (**B**). Lines are ordered along the x-axis according to total organic acid concentration

**Fig. 5:**
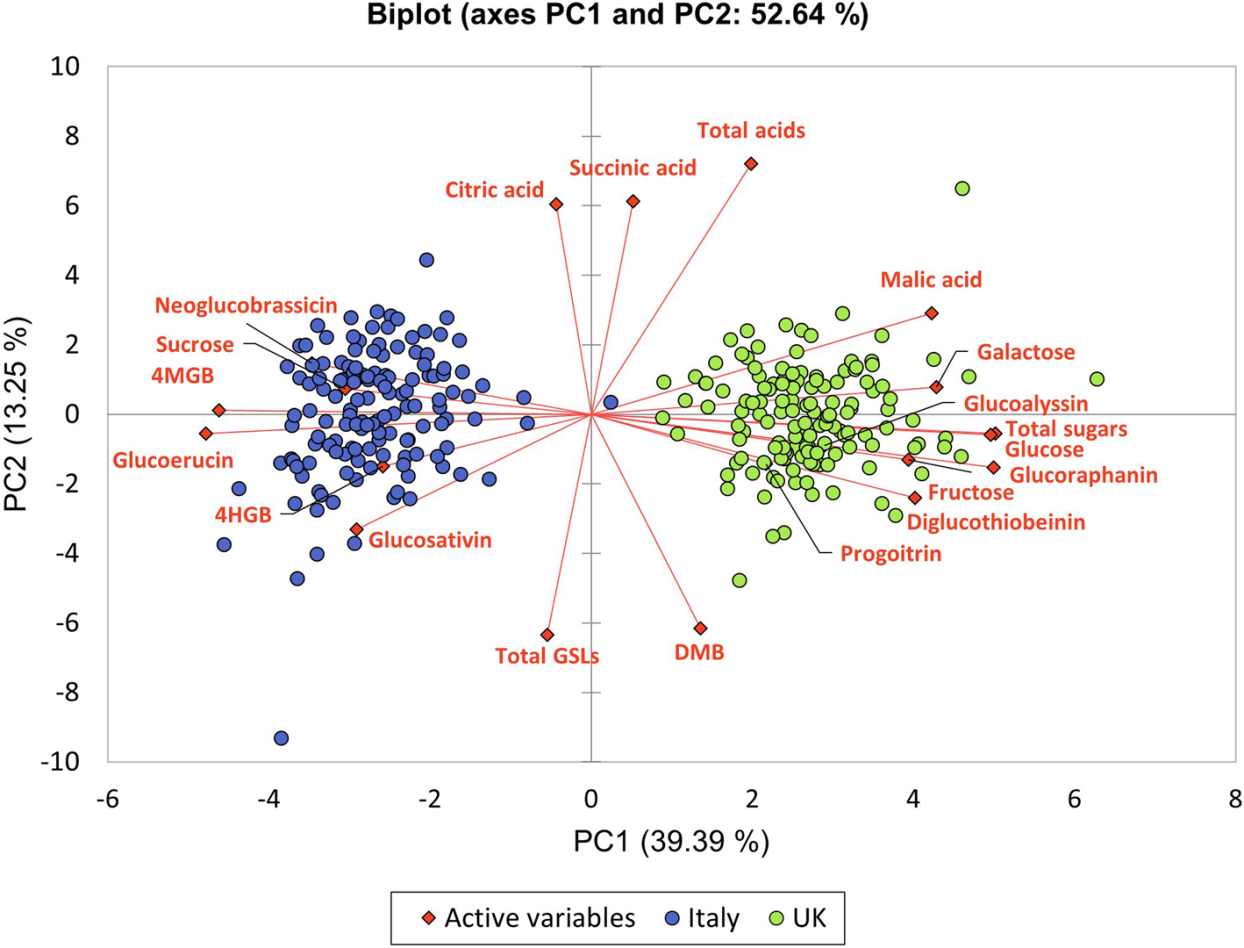
Principal Component Analysis biplot representing 52.64% of metabolite variance across the *Eruca vesicaria* subsp. *sativa* mapping populations grown in Italy and the UK. PC1 and PC2 explain 39.9 and 13.25% of the total variation between samples, respectively. Eigenvalues for PC1 and PC2 were 7.88 and 2.65, respectively. Abbreviations: DMB, dimeric 4-mercaptobutyl GSL; 4HGB, 4-hydroxyglucobrassicin; 4MGB, 4-methoxyglucobrassicin

Composition data for GSLs are presented in Fig. 2, for sugars in Fig. 3, and organic acids in Fig. 4. Accompanying ANOVAs (Analysis of Variance) with post hoc Tukey’s HSD (Honestly Significant Difference) test statistical summaries are provided in Additional File 1. A Principal Component Analysis (PCA) of the metabolite data was performed, with the biplot of variable separation presented in Fig. 5. PC1 and PC2 were selected for presentation as they accounted for the greatest amount of explained variation between samples (52.64%).

Individual GSL compound concentrations varied significantly between Italy and UK trials. UK-grown plants were characterized by significantly higher average GRA (3.3 mg g^− 1^ dw; *p* = < 0.0001), progoitrin (PRO, 0.2 mg g^− 1^ dw; *p* = < 0.0001), glucoalyssin (GAL, 0.2 mg g^− 1^ dw; *p* = < 0.0001), DGTB (0.2 mg g^− 1^ dw; *p* = < 0.0001), and DMB (13.9 mg g^− 1^ dw; *p* = < 0.0001) concentrations. Italy-grown plants by contrast contained significantly higher average concentrations of GSV (0.6 mg g^− 1^ dw; *p* = < 0.0001), 4-hydroxyglucobrassicin (4HGB, 0.03 mg g^− 1^ dw; *p* = < 0.0001), GER (2.8 mg g^− 1^ dw; *p* = < 0.0001), 4MGB (2.6 mg g^− 1^ dw; *p* = < 0.0001), and NGB (0.4 mg g^− 1^ dw; *p* = < 0.0001). PCA (Fig. 5) revealed a distinct separation between Italy and UK-grown plants, with separation along PC1 driven by total sugars and the specific GSLs mentioned above, with the UK separating on the right, and Italy on the left. The significant differences observed between the two trials (Additional File 1) suggest a clear impact of growth environment upon GSL profile compositions, but not total concentrations, driving separation of the data (Fig. 5). Of note is the disparity between indolic compound concentrations in Italy-grown plants versus the UK (Fig. 2 and Fig. 5). Concentrations of NGB and 4MGB were 1.6-fold and 5.6-fold greater on average in the Italian trial, respectively. By contrast the UK plants contained 2.3-fold higher concentrations of GRA, on average.

Several compound concentrations were found to segregate significantly (Additional File 1). In the Italy trial GRA concentrations ranged between 1 mg g^− 1^ dw and 2.3 mg g^− 1^ dw (*p* = < 0.0001; Fig. 2). This contrasts with concentrations in the UK, which ranged from 2 mg g^− 1^ dw to 4.9 mg g^− 1^ dw (*p* = < 0.0001). This trend was not seen for other aliphatic GSLs. For example, GER concentrations varied significantly in Italy (1.8 mg g^− 1^ dw to 4 mg g^− 1^ dw; *p* = < 0.0001), but not in the UK. Also of note is that concentrations of GSV did not vary significantly within each respective trial, but concentrations of its dimeric form (DMB) did. In Italy, DMB concentrations ranged from 8 mg g^− 1^ dw to 23.5 mg g^− 1^ dw (*p* = < 0.0001), and in the UK 9.2 mg g^− 1^ dw to 19.6 mg g^− 1^ dw (*p* = 0.001). The indolic GSL 4MGB had a wide range of concentrations across the population, being observed as low as 0.04 mg g^− 1^ dw in the UK and 0.7 mg g^− 1^ dw in Italy, and as high as 2.5 mg g^− 1^ dw in the UK and 6.5 mg g^− 1^ dw in Italy (*p* = < 0.0001 and 0.042, respectively). The related indolic GSL, NGB, occurred in lower concentrations, ranging between 0.2 mg g^− 1^ dw and 0.9 mg g^− 1^ dw in Italy (*p* = < 0.0001), and 0.01 mg g^− 1^ dw and 0.7 mg g^− 1^ dw in the UK (*p* = < 0.0001).

Plants grown in the UK contained twice as much total sugar (80.2 mg g^− 1^ dw) as those grown in Italy (39.6 mg g^− 1^ dw; *p* = < 0.0001), on average (Fig. 3). This is reflected in the individual sugar components: average glucose, galactose and fructose concentrations in the UK were 2.4-fold (61.2 mg g^− 1^ dw), 1.8-fold (5.2 mg g^− 1^ dw), and 2.1-fold (11.5 mg g^− 1^ dw) higher than Italy, (25.5 mg g^− 1^ dw, 3 mg g^− 1^ dw, and 5.4 mg g^− 1^ dw) on average, respectively (Fig. 3). The only exception was for sucrose, which had the highest average concentrations in Italy-grown plants (5.7 mg g^− 1^ dw), 2.4-fold higher than the UK (2.3 mg g^− 1^ dw). These differences can be observed in PCA data (Fig. 5) where total sugars, glucose and fructose concentrations are associated with the UK sample cluster (green circles), and sucrose with the Italy cluster (blue circles). UK trial plants had significantly different glucose, fructose, and total sugar concentrations between lines (*p* = < 0.0001, 0.012, and 0.000, respectively). No significant differences were observed for individual sugars in Italy, suggesting that total sugar accumulation in rocket leaves has a predominantly environmental component.

Variation in organic acids was not predominantly driven by region, as shown in Fig. 5. Whereas there were no significant differences in organic acids between lines grown in the UK, there were differences between the Italian lines. Average Italy-grown concentrations ranged from 59.7 mg g^− 1^ dw to 147 mg g^− 1^ dw (*p* = 0.001) for citric acid, and 19 mg g^− 1^ dw to 83.8 mg g^− 1^ dw (*p* = < 0.0001) for malic acid (Additional File 1, Fig. 4). On average there was no significant difference between the UK and Italy trials for succinic acid, but a significantly higher average concentration of malic acid was observed in the UK (65.4 mg g^− 1^ dw; *p* = < 0.0001). This produced an overall significant difference in total organic acids for the UK trial (262 mg g^− 1^ dw) versus the Italy trial (243.5 mg g^− 1^ dw; *p* = < 0.0001) on average. The abundance of malic acid in UK-grown samples showed strong separation along PC1 (Fig. 5) and is closely associated with the UK PCA sample cluster (green circles).

Lines within the population displayed an environment-dependent response in concentrations of the three metabolite classes measured. Additional File 2 contains a ranking of the 139 lines (and their parents) grown in each environment for total GSL, total sugar, and total organic acid concentrations. Very few lines displayed consistency and trait stability between Italy and UK trials. Only 19 lines contained similar total GSL concentrations in both trials. Several lines displayed differential accumulations between the trials. The most striking example is line 85, which contained the highest total GSL concentrations in the Italy trial, but only 112th highest in the UK. No lines were found to have comparable total sugar concentrations between trials. Only two lines (26 and 141) had higher sugar accumulation in Italy than in the UK. Total organic acid concentrations were uniform between trials for only 13 lines. While organic acid concentrations were less variable between the trials, a notable exception was line 121 which had highest total concentrations in the Italy trial, but only the 121st highest in the UK. Conversely, line 80 contained the highest concentrations in the UK trial, and third lowest (139th) in the Italy trial.

### SNP linkage map

From a total of 709 potential markers 453 candidates showed sufficient polymorphism in the F_4_ recombinant inbred line (RIL) mapping population available in this study. Of these JoinMap 4 (Kyazma, Wageningen, Netherlands) created a final map of 285 markers distributed over 18 linkage groups (for details of SNP selection criteria see the Methods section). The reported linkage groups (LGs) have an average distance between markers of 3.1 cM (Table 1). The data indicate that the map is robust and has parity with previously published maps of other Brassicaceae species (Qu et al. 2016).

**Table 1:** *Eruca vesicaria* subsp. *sativa* draft linkage map statistics

Linkage group	Map length (cM)	Number of markers	Average distance between markers (cM)
1	84.5	7	12.1
2	21.6	9	2.4
3	47.3	37	1.3
4	15.3	11	1.4
5	27.2	23	1.2
6	89.0	23	3.9
7	27.7	10	2.8
8	30.4	28	1.1
9	39.7	19	2.1
10	53.1	12	4.4
11	11.3	11	1.0
12	95.0	11	8.6
13	74.8	11	6.8
14	38.0	9	4.2
15	46.4	9	5.2
16	96.9	32	3.0
17	33.5	14	2.4
18	57.5	9	6.4
Total (average)	889.2	285	(3.1)

**Fig. 6:**
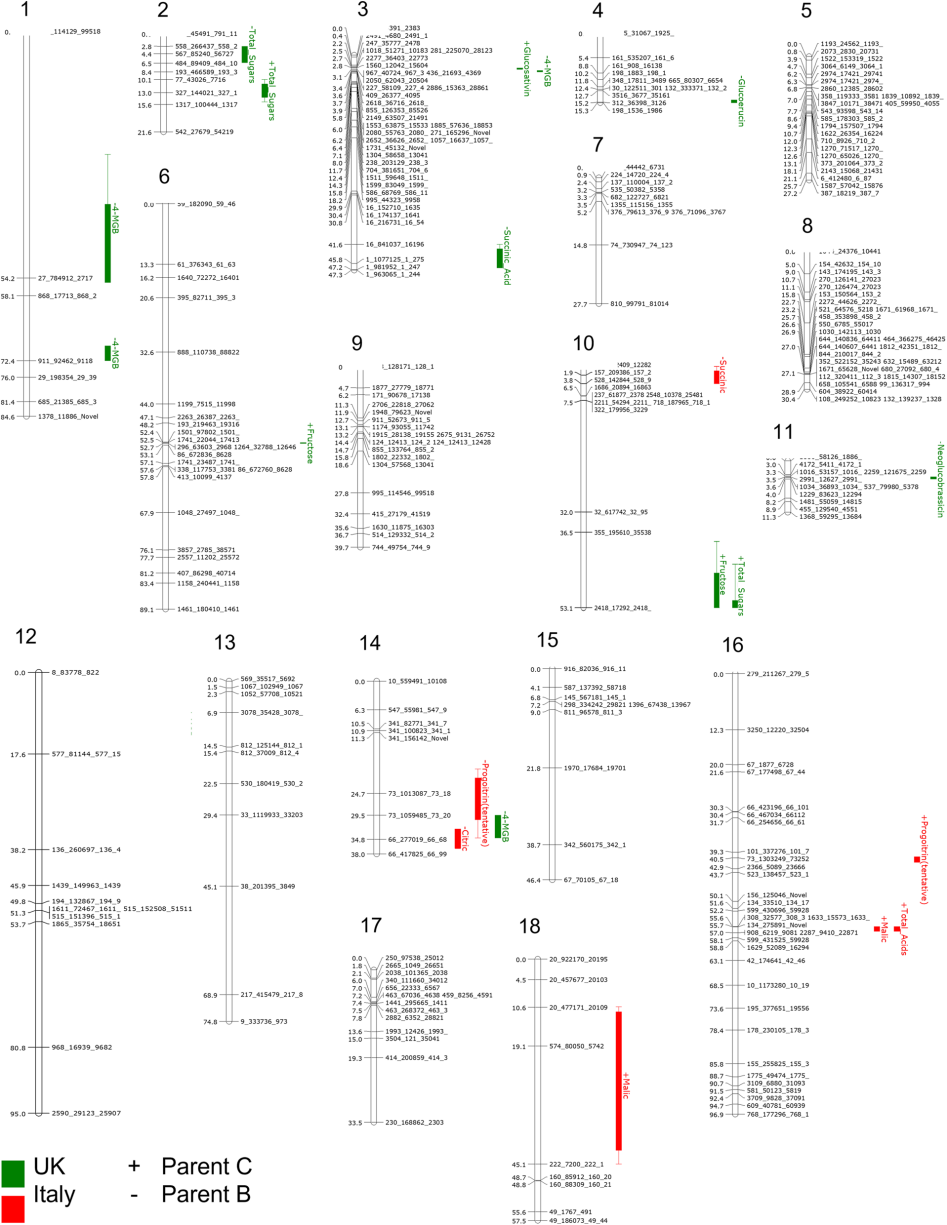
QTL map showing marker distances on all *Eruca vesicaria* subsp. *sativa* linkage groups, as well as the location of QTLs identified from field trials in the UK and Italy (see inset). +/− symbols represent higher content of a given compound arising from parent C allele or the parent B allele, respectively

The linkage map produced 18 LGs of varying density and length (Table 1; Fig. 6). *Eruca* contains 11 pairs of chromosomes (2*n* = 22) (Bell and Wagstaff 2019) indicating that the distribution and density of selected SNPs was not sufficient to resolve chromosome-equivalent LGs. Several identified LGs may be representative of single chromosomes due to the presence of markers from common reference genome assembly scaffolds (Bell et al. 2020a; Additional File 3). LG1 is low density compared to LG3 and LG9, containing only seven markers. LG2 and LG6 both contain markers from scaffold 193 and based on scaffold information, we hypothesize that they represent a single contiguous LG. Likewise, LG4 and LG8, and LG14, LG15, and LG16 (Fig. 6) may belong to the same respective groups for this same reason, but increased marker density will be required to verify this.

### QTL map for glucosinolate, sugar, and organic acid compositions

QTL data for the analyzed metabolites are presented in Table 2 with LOD scores, position, confidence intervals, and explained variation percentages. Of the QTL identified for Italy and UK trials, none for the same traits overlapped between countries (Fig. 6). This indicates that there is a strong genotype x environment (GxE) interaction determining the abundance of both primary and secondary metabolites in rocket. An ANOVA of the metabolite data (Additional File 4) confirmed that GxE significantly influenced GRA (*p* = < 0.0001), GAL (*p* = 0.013), DMB (*p* = 0.005), NGB (*p* = < 0.0001), total GSL concentrations (*p* = 0.004), sucrose (*p* = 0.006), and malic acid (*p* = 0.002). Environmental effects alone significantly contributed to differences in all metabolite concentrations, apart from succinic acid. When only genotype is considered, significant differences between *Eruca* lines were observed for all metabolites except DGTB, GSV, sucrose, fructose, total sugars, and succinic acid.

**Table 2:** Quantitative Trait Loci statistics for glucosinolates, sugars, and organic acids identified and quantified in a rocket mapping population (*n* = 139) grown in Italy and the United Kingdom

Compound	Linkage group	Nearest marker	Marker position (cM)	QTL interval (cM)	LOD score	Explained variance (%)	Additive effect
Italy trial							
*Glucosinolates*							
Progoitrin	14	73_1013087_73.188	25.75	20.70	3.52	20.0	−0.04
Progoitrin	16	73_1303249_73.252	40.52	1.21	2.90	16.0	0.03
*Organic acids*							
Succinic acid	10	157_209386_157.28	2.94	3.77	2.84	9.1	−5.78
Citric acid	14	66_277019_66.68	34.79	4.27	4.44	13.9	−21.75
Malic acid	16	134_275891_Novel00420	55.67	1.07	3.59	9.6	3.57
Total organic acids	16	134_275891_Novel00420	55.67	1.07	2.97	9.4	10.43
Malic acid	18	574_80050_574.2	23.11	28.51	4.75	15.8	−4.67
UK trial							
*Glucosinolates*							
4MGB	1	27_784912_27.17	48.00	33.22	3.20	23.3	−0.29
4MGB	1	911_92462_911.8	71.10	2.31	4.90	38.7	−0.94
Glucosativin	3	967_40724_967.3	3.06	0.36	3.86	11.8	0.04
4MGB	3	409_26377_409.5	3.55	0.30	4.63	13.1	−0.14
Glucoerucin	4	198_1536_198.6	15.29	0.57	3.16	9.8	−0.10
NGB	11	537_79980_537.8	3.65	0.44	3.12	9.7	0.03
4MGB	14	73_1059485_73_20	29.52	5.00	2.94	10.3	−0.13
*Sugars*							
Total sugars	2	567_85240_567.27	4.39	3.57	6.05	16.6	−8.49
Total sugars	2	327_144021_327.19	13.01	4.94	3.12	8.2	5.96
Fructose	6	296_63603_296.8	52.72	0.25	3.63	10.2	1.08
Fructose	10	2418_17292_2418.2	53.11	17.11	2.93	8.1	0.95
Total sugars	10	2418_17292_2418.2	53.11	14.60	2.91	7.6	4.33

*Abbreviations*: *4MGB* 4-methoxyglucobrassicin, *NGB* Neoglucobrassicin

Five QTL were identified for indolic GSLs (4MGB and NGB) within the UK trial (Table 2). Broad QTL were found for 4MGB on LG1 (33.2 cM) and LG14 (6.04 cM; Table 2 and Fig. 6), which is reflective of low marker density in these regions. The QTL identified on LG3 was much narrower, however (0.03 cM), and is collocated with a locus for GSV (UK), an aliphatic GSL. A QTL for NGB in the UK trial was found on LG11. The only other GSL QTL observed for the UK-grown population was GER on LG4. QTL for PRO were found in the Italy-grown plants on LG14 and LG16. This further corroborates the hypothesis that these belong to a single LG due to their co-location over scaffold 73. Of QTL for GSLs, all but one (PRO, LG16) had LOD scores > 3.0, indicating a low probability (*p* = < 0.05) that the markers are associated by chance (Risch 1991).

QTL for total sugar and fructose concentrations were identified on LG2 and LG10 based on the UK population data. The latter of these on LG10 co-locate (at 53.1 cM), indicating a possible shared locus for sugar regulation (Table 2, Fig. 6). An additional QTL for fructose concentration was present on LG6 with a narrow interval of 0.3 cM. No sugar-related QTL were observed for the Italy-grown population, but several loci for organic acids were identified. These were found on LG10 (succinic acid), LG14 (citric acid), and LG18 (malic acid). Of note is an additional co-locating locus for malic and total organic acids on LG16 (at 55.7 cM). This is suggestive of a strong underlying marker for these metabolites and that total organic acid concentrations are driven by malic acid. An additional QTL for succinic acid was identified on LG3 for the UK trial. All the sugar and organic acid markers (except for LG10 total sugars and fructose) had LOD scores > 3.0, indicating a statistically significant (*p* = < 0.05) association between traits and SNP markers.

### Genes underlying identified QTL

Where QTL were identified, the underlying genome assembly scaffolds were scrutinized for transcriptional or biosynthetic genes related to the respective metabolites. This was done through utilization of a genome annotation (Bell et al. 2020a) and visualization using Integrated Genomics Viewer (IGV; Robinson et al. 2017).

Genes related to GSL metabolism are presented in Table 3 with details of their DNA sequence similarities with related Brassicaceae. DNA and protein sequence species BLAST scores are provided in Additional File 5 and Additional File 6, respectively. Individual gene protein sequence alignments between closest matching species are provided in Additional File 7. The high percentage similarity of *Eruca* gene sequences with other species indicates that these genes are likely to be orthologous and may have the same or similar function(s).

**Table 3:** Genes identified underlying 4-methoxyglucobrassicin QTL with the closest match NCBI accession sequences from related species

Linkage Group	Scaffold number	Gene annotation^a^	Closest species match (BLAST)	Gene ID	BLAST query cover %	Identity %	NCBI accession
1	27	*MYB51*	*Eruca vesicaria* subsp. *sativa*	*MYB51*	61%	99.8%	JX946185.1
			*Brassica napus*	*MYB51*	99%	87.3%	XM_013871637.2
			*Brassica oleracea*	*MYB51*	95%	87.6%	XM_013749453.1
1	27	*IGMT1*	*Raphanus sativus*	*IGMT2*	99%	92.6%	XM_018606705.1
			*Brassica rapa*	*IGMT4*	100%	92.4%	XM_009111947.3
			*Brassica oleracea*	*CCOAOMT*	100%	92.3%	XM_013747047.1
1	27	*IGMT4*	*Brassica rapa*	*IGMT4*	96%	91.0%	XM_009111948.3
			*Raphanus sativus*	*IGMT4*	89%	92.6%	XM_018606707.1
			*Brassica napus*	*IGMT4*	96%	89.8%	XM_013866689.2
12	8	*TIFY 11A* (*JAZ5*)	*Brassica napus*	*TIFY 11A* (*JAZ5*)	86%	85.5%	XM_013827518.2
			*Brassica rapa*	*TIFY 11A* (*JAZ5*)	88%	86.5%	XM_009112165.3
			*Brassica oleracea*	*TIFY 11A* (*JAZ5*)	84%	87.0%	XR_001263608.1
12	8	*TSB-like*	*Brassica napus*	*TSB1*	100%	93.2%	XM_013801988.2
			*Brassica rapa*	*TSB1*	100%	93.1%	XM_009112467.3
			*Raphanus sativus*	*TSB1*	100%	92.5%	XM_018594129.1

^a^ = Bell et al. 2020a

*Abbreviations*: *QTL* Quantitative trait loci, *4MGB* 4-methoxyglucobrassicin, *IGMT* Indole glucosinolate methyl transferase, *CCOAOMT* Caffeoyl-CoA O-methyltransferase, *JAZ* Jasmonate ZIM domain-containing, *TSB* Tryptophan synthase beta chain

LG1 contained SNPs located on genome assembly scaffold 27 (position 48 cM, interval 33.2 cM; Table 2). Analysis of the underlying annotated genes (Bell et al. 2020a) revealed three related to indole GSL biosynthesis – *MYB51*, *IGMT1*, and *IGMT4* (*Indole Glucosinolate Methyl Transferase 1* and *4* isoforms; Additional File 3). *MYB51* is known to be the primary transcription factor modulating indole GSL biosynthesis in *Arabidopsis* (Gigolashvili et al. 2007), and IGMT1 and IGMT4 are *O*-methyltransferases responsible for methoxylation of GBC to form 4MGB (Ku et al. 2016; Rahikainen et al. 2017). Another narrower 4MGB QTL is located on LG1 (position 71.1 cM, interval 2.3 cM; Table 2) over scaffold 911. 13 genes are annotated on this scaffold and a further seven are hypothetical or of unknown function (Additional File 3). None of the annotated genes are thought to be linked with indolic GSL biosynthesis, however this may be a region of interest to explore novel gene functions in *Eruca*.

Two QTL are present on LG3: one for GSV (position 3.06 cM, interval 0.36 cM), and another for 4MGB (position 3.55 cM, interval 0.3 cM; Table 2). The proximity of these is indicative of a gene involved in regulating both aliphatic and indolic GSLs. Analysis of the underlying scaffolds (1560, 967, and 436) revealed no genes in the annotation known to be associated with this function, however there are 11 putative genes of unknown function present which may be worthy of further investigation at this locus (Additional File 3).

Two genes related to indole GSL biosynthesis and jasmonate (JA) response were present on LG12, scaffold 8 (position 0 cM, QTL interval 21.6 cM), which underlies an additional QTL for 4MGB. These genes were putatively identified in the annotation as *JAZ5* (*JAZMONATE ZIM-DOMAIN PROTEIN 5*; also known as *TIFY 11A*) and a *TSB-like* (*Tryptophan Synthase Beta chain-like*) gene (Bell et al. 2020a). JAZ proteins interact with MYC2 (Chini et al. 2016) and are therefore related to GSL metabolism via MYB transcription factors such as *MYB51*, which are in turn regulated by MYC-encoding genes. *TSB1* encodes an enzyme for tryptophan biosynthesis, which is the amino acid precursor to 4MGB and other indole GSLs (Gigolashvili et al. 2007).

### Amino acid sequence differences between parental genotypes

Three SNPs were found within the identified *JAZ5* ortholog (Additional File 8). Two SNPs produce non-conservative mutations in the amino acid sequence at positions 45 (proline to serine) and 127 (glutamic acid to valine). The TIFY domain (TIF[F/Y]XG; Vanholme et al. 2007) is highly conserved between species (Additional File 8) and these changes are unlikely to alter structure or function of the protein. This suggests that its function is likely to be the same as that reported for model species (Jin and Zhu 2017). SNPs were identified in *IGMT1* and *IGMT4* and give rise to conservative mutations at residue 18 (isoleucine to valine) and residue 307 (glutamic acid to aspartic acid), respectively.

Analysis of the *MYB51* sequence revealed multiple SNPs creating changes in the amino acid sequence (Additional File 8). Two non-conservative changes are present, flanking a region of low complexity (Simple Modular Architecture Research Tool, SMART: https://smart.embl.de/smart/show_motifs.pl?ID=A0A178WB71_ARATH; Letunic and Bork 2018), at residues 208 (glutamine to proline) and 229 (serine to proline). Another conservative mutation is present at residue 231 (leucine to phenylalanine), and a semi-conservative change at 332 (asparagine to serine). No SNP changes are in proximity to the two DNA-binding domains (DBDs; positions 72 to 123, and 126 to 174) but the changes at positions 229 and 231 are near the highly conserved MYC-interaction motif (MIM; [F]LN[R][V]A). The core xLNxxA motif is required for binding with bHLH (basic helix-loop-helix) MYC transcription factors, such as MYC2, MYC3 and MYC4, which are responsible for the interaction of GSL transcription factors with the JA signaling pathway (Millard et al. 2021). It is therefore likely that the identified ortholog of *MYB51* is associated with the regulation, biosynthesis, and metabolism of 4MGB in *Eruca*.

## Discussion

### Study limitations

The main limitation of the presented study is the relatively low SNP density within the linkage map, and chromosome-level LGs could not be fully resolved. LGs of comparatively high density (e.g., LG3, LG5, and LG8) were generated, and others with few markers (e.g., LG1, LG10, LG12, and LG13), indicating a lack of coverage in some regions. Despite the low mapping density, several loci associated with GSL, sugar, organic acid metabolism have been identified. Future iterations that include greater SNP density will assist in improving the map and resolving the identities of LGs into corresponding chromosomes, as has been achieved with other vegetable crop species (Iorizzo et al. 2016).

### UK-grown *Eruca* is characterized by high glucoraphanin, monosaccharide, and malic acid concentrations

The largest differences in metabolic profiles between the two trial locations were seen in the health related GSL GRA, monosaccharides (glucose, galactose, and fructose), and malic acid concentrations (Fig. 5), which were all more abundant in the UK than Italy. GRA is the precursor to the ITC sulforaphane (SF), with its beneficial health effects being well established (Neequaye et al. 2022); therefore identifying lines that produce GRA in high abundance under UK growing conditions is useful for breeding programs targeting nutrition. For sugars a similar trend was previously reported by Bell et al. (2020b) in ‘wild’ rocket (*Diplotaxis tenuifolia*), where UK grown crops had higher GRA concentrations than those from Italy. Higher sugar and malic acid concentrations in the UK may be connected to plant stress because of exposure to suboptimal climate conditions, and perhaps increased pest and disease burdens. ‘Salad’ rocket is a species native to the warm and dry climates of the Mediterranean basin, Middle East, and Pakistan, and has evolved under warm dry conditions (Westberg et al. 2013). As such, the cool, wet, and humid climate of the UK is potentially stressful and results in lower quality leaves (Bell et al. 2020b). Plants produce ATP by oxidizing reduced sugars (galactose, glucose, and fructose) through respiration (Bisbis et al. 2018), and high respiration rates in salad crops are associated with increased oxidative stress and reduced postharvest quality (Ripoll et al. 2019). A reason for the significantly higher amounts of sucrose in Italian-grown plants may be because it has not been hydrolysed to form glucose and fructose monosaccharides, which were significantly lower in concentration compared to the UK trial. In UK plants (under greater oxidative stress) we hypothesize that sucrose would be hydrolysed to produce greater amounts of monosaccharides, making them available to produce more ATP and sustain cellular processes. Cytosolic proteins involved with organic acid metabolism are known to coordinate concentrations of malate in response to stress (Ludwig 2016), and in combination with higher sugar concentrations may afford plants a mechanism of tolerance to the UK cultivation environment. This hypothesis may explain both the high malic acid and sugar concentrations found in the UK-grown population.

### *Eruca* glucosinolate profiles are significantly influenced by cultivation environment

Our hypothesis that Italy-grown plants would contain higher concentrations of total GSLs cannot be accepted based on the observations. The picture of GSL composition between environments appears to be much more subtle, with concentrations of individual compounds affected by the environment rather than the total abundance, on average. Previous research of temperature effects on *A. thaliana* (Kissen et al. 2016) has shown that cooler temperatures promote aliphatic GSL biosynthesis, but that total concentrations are genotype dependent. Similar effects were observed in this study, with aliphatic GSLs such as GRA and GAL significantly higher in the comparatively cooler UK trial (Fig. 2, Fig. 5). The effect was not observed for all aliphatic GSLs, however; the opposite trend was true for GSV and GER, where average concentrations were higher in the Italy trial.

A significant differential accumulation pattern was observed for GSV and DMB, further adding evidence to the hypothesis that accumulations of the monomer and dimer forms are GxE dependent (Additional File 4). DMB concentrations were significantly higher in the UK climate, which supports previous observations of commercially grown ‘salad’ rocket and ‘wild’ rocket leaves (Bell et al. 2020b).

### Genes underlying QTL for 4-methoxyglucobrassicin are suggestive of interaction with MYC2

*MYB51* is the primary regulator of indolic GSL biosynthesis and its identification under a QTL for 4MGB makes it a strong candidate for further investigation. Its proximity to two IGMT gene isoforms also indicates that this is a locus for both transcriptional regulation and synthesis of the compound. MYB51 is known to interact directly with MYC2 via a MIM, which is conserved between all known GSL-regulating MYB transcription factors (Millard et al. 2021). The presence of a *JAZ5* (*TIFY 11A*) gene underlying a separate locus for 4MGB on LG12 indicates that JA signaling may influence concentrations via interaction with MYC2. The interaction between JAZ proteins, MYCs and MYBs is well known in *Arabidopsis* (Chini et al. 2016; Additional File 9), and the mechanisms for regulating GSL concentrations have been demonstrated in the model plant. *A. thaliana myc2/3/4* mutants are severely impaired in their ability to synthesize GSLs in response to mechanical wounding stress (Schweizer et al. 2013), for example.

The picture of indolic GSL biosynthesis and control in rocket is complicated by the fact that gene duplication has occurred (Bell et al. 2020a). Three copies of *MYB51*, two copies of *IGMT1*, three copies of *IGMT4*, two copies of *TSB1*, three copies of *MYC2*, and three copies of *JAZ5* (*TIFY 11A*) have been putatively identified in the genome annotation. Gene expression data of parent lines of the population used in this study (Bell et al. 2020a) have shown significant differential expression patterns of each respective gene copy, and the level of co-expression between copies is not uniform, particularly for *MYC2* and *MYB51*. It is unknown if all the identified copies are redundant or indeed functional within *Eruca*. Expression profile data of the parent lines (Bell et al. 2020a) suggests that that the dominance of expression of specific gene copies may be genotype dependent.

We have identified novel genetic loci in *Eruca* for GSL, sugar, and organic acid content for the first time. This information can be utilized by breeders to modify metabolite composition and has brought about new understanding of how plants respond on a phytochemical level to contrasting cultivation environments.

## Methods

### Plant material

139 lines of F_4_ *E. sativa* plants were produced from a biparental cross of homozygous parents (self-pollinated for five generations) at Elsoms Seeds Ltd. (Spalding, UK). F_1_ seed produced from the cross were self-pollinated in controlled glasshouse conditions to generate the F_4_ seed. The inbreeding coefficient for individuals in the population was calculated to be 0.938 (Falconer and Mackay 1996), with an estimated residual heterozygosity of 6.2%.

The mapping population was grown in two locations reflective of commercially produced rocket: a polytunnel near Rome, Italy (41°55′31.1″N 12°08′15.8″E) in September 2017, and in open field near Owermoigne, Dorchester, UK (50°40′40.9″N 2°19′34.3″W) in June 2018. For both trials, ten seeds of each line, per experimental block, were sown into peat blocks and covered with vermiculite. Parent lines (designated B and C) were included in each trial giving a total of 141 lines. Seeds were germinated and raised for 20 days in a vented glasshouse at Elsoms Seeds before being transported by temperature-controlled van (10 °C) to the respective trial sites. Upon arrival seedlings were transplanted by hand into soil in parallel rows stretching the width of a 1.5 m bed with 10 cm spacing between rows. Plant lines were marked and identified using colored stakes and labels adjacent to each individual row. In the Italian trial two adjacent beds were used and all plants were contained in a single tunnel span. In the UK trial seedlings were planted in a single continuous length of field. Each trial comprised a complete randomized block design of three replicates. Each block was surrounded by a commercial ‘salad’ rocket guard (provided by Elsoms Seeds) to provide a buffer against edge effects.

The average daily temperature for the duration of the growth period post-transplantation (14 days) was 22.4 °C in Italy, and 14.9 °C in the UK. The average daily maximum and minimum for the period in Italy was 24.2 °C and 20.7 °C, respectively. In the UK average daily temperature maximum and minimum was 16 °C and 13.6 °C, respectively. Average cloud cover percentage in Italy for the duration of the trial was 21.1%, and in the UK 53.1%. Relative humidity in Italy was an average of 68.2%, and in the UK 86.4%. The UK crop received 2.1 mm of rainfall across the 14-day trial period. This was supplemented with overhead spray irrigation to maintain trial viability. In the Italy polytunnel environment plants received daily overhead irrigation, as per standard industry practice.

Rocket plants were harvested in the morning after 14 days of growth in each respective trial. Leaves of one plant from each line and block were harvested and placed into plastic bags (*n* = 3). These samples were utilized for DNA extractions and SNP genotyping. A further four plants from each line were harvested and pooled to give a representative leaf sample for phytochemical analyses. This was done separately for each block giving *n* = 3 pooled samples per line, and 423 samples in total. Immediately after harvest, bags of leaves were placed into crates and stored in a nearby cold room in Italy, and a temperature-controlled van in the UK (both 4 °C). Samples from both locations were driven in a temperature-controlled van (4 °C) to the University of Reading School of Chemistry, Food and Pharmacy (Reading, UK). The differences in transit duration are consistent with comparable supplies of rocket leaves from each location (~ 36 hours transit from Italy, and ~ 2 hours transit within the UK). Upon arrival crates were placed directly into a − 20 °C freezer room until further processing for metabolite analysis and DNA extraction.

### Metabolite analyses

Prior to extraction all samples were lyophilized in batches for three days. Dried material was then ground into a fine powder using a Wiley Mini Mill (Thomas Scientific, Swedesboro, NJ, USA).

Intact GSLs were extracted and analyzed by LC-MS according to the method presented by Jasper et al. (2020). Authentic standards were used to generate external calibration curves (purchased from PhytoPlan, Heidelberg, Germany). Standards were prepared according to the protocol of Jin et al. (2009): GRA (99.86%; *r*^*2*^ = 0.997), progoitrin (PRO; 99.07%; *r*^*2*^ = 0.998), glucoalyssin (GAL; 99.8%; *r*^*2*^ = 0.999); 4-hydroxyglucobrassicin (4HGB; 96.19%; *r*^*2*^ = 0.992), GER (99.68%; *r*^*2*^ = 0.999), GBC (99.38%; *r*^*2*^ = 1). All compound purities were determined by HPLC Diode Array Detector (DAD). DGTB, GSV, and DMB were semi-quantified using GER, and 4MGB and NGB were semi-quantified using GBC, as authentic standards were unavailable. GLSs were identified according to their parent ion *m/z* as previously reported by Jasper et al. (2020).

For sugars and organic acid extraction, 100 mg of lyophilized material was added to 10 mL of 10 mM hydrochloric acid in glass vials. Magnetic stirrers were used to mix samples at ambient temperature for 30 mins. 1 mL aliquots of the slurry were removed and centrifuged in 1.5 mL tubes for 15 minutes at 12.5 x *g*. Supernatant was removed and filtered through 0.22 μm PVDF filters (Cole Palmer, St. Neots, UK). Samples were run on an Agilent 1260 Infinity II system with quaternary pump, autosampler, degasser, column oven, DAD, and refractive index (RI) detectors. Compounds were separated on an Aminex HPX87H column (BioRad, Watford, UK) with an isocratic gradient of 5 mM sulfuric acid at a flow rate of 0.3 mL min^− 1^. Sugars were quantified using the RI detector, with the flow cell purged after every ten sample runs. Organic acids were quantified using the DAD detector at a wavelength of 190 nm. Authentic standards for sugars and organic acids were purchased from Merck-Sigma (Gillingham, UK) and prepared in a concentration range of 0.01–1 mg mL^− 1^: fructose (> 99%; *r*^*2*^ = 0.999), galactose (> 99%; *r*^*2*^ = 1), glucose (> 99%; *r*^*2*^ = 1), sucrose (> 99%; *r*^*2*^ = 0.996), citric acid (> 99%; *r*^*2*^ = 1), malic acid (> 99%; *r*^*2*^ = 1), succinic acid (> 99%; *r*^*2*^ = 1).

### Statistical analyses

All metabolite statistical analyses were performed using XL Stat (Addinsoft, Paris, France). Shapiro-Wilk normality tests were performed on each variable and were found to fit a normal distribution. Protected ANOVA tests were conducted to determine both within and between trial variation of each rocket line, as well as GxE effects. Post hoc Tukey’s HSD tests were applied for multiple pairwise comparisons between individual plant lines and environments (e.g., Italy vs. UK; Additional File 1 and Additional File 4). PCA was performed using Pearson’s correlation coefficient analysis, with *n-1* standardization, and Kaiser Normalization. Variables were grouped within the analysis according to the field trial country.

### Single nucleotide polymorphism genotyping

SNPs were identified between transcriptomes of parent lines B and C (Bell et al. 2020a), performed by Novogene (HK) Co. Ltd. (Hong Kong) as a service. Transcripts from line B were compared to C, which is the reference genome for ‘salad’ rocket (Bell et al. 2020a). Samtools was used sort reads according to genome co-ordinates, with screening and removal of repeated reads (Li 2011). GATK2 was used to conduct variant calling (Van der Auwera et al. 2013). 703 SNPs were selected based on their quality scores (Phred > 60; *Q* = − 10 log_10_ *p*) to generate the genetic linkage map. DNA was extracted from the collected mapping population samples using DNeasy Plant Kits (Qiagen, Manchester, UK) according to the manufacturer protocol. Extracted samples were sent to Bejo Zaden BV (Warmenhuizen, The Netherlands) for genotyping according to the protocol of van Haperen et al. (2020). Kompetitive allele specific PCR (KASP) primers for selected SNPs are provided in Additional File 10.

### Linkage map construction and QTL analysis

After screening for and removing highly heterozygous SNPs and those which were heavily skewed (> 90% of lines expressing a single allele), the 453 remaining were entered into JoinMap 4 (van Ooijen 2006) to build a map. Of these 302 formed 18 linkage groups and 285 were included in the final map. The remainder did not form enough statistically significant linkages within the group to be placed within the map. 17 markers were removed as they collocated to other markers or lacked sufficient recombination for the algorithm to generate a reliable order. Groupings were formed using the ‘recombination frequency’ parameter, and Haldane’s mapping function (Logarithm of Odds threshold 0.01, recombination frequency threshold 0.49, jump threshold 5.0, ripple value 1) was used to generate map orders.

Predicted means from three biological repeats of each metabolite analysis were generated by ANOVA within XLStat (Addinsoft) and were subsequently used in QTL mapping. LOD scores were determined using 5000 permutations, and a significance threshold (*p* = < 0.05) was determined from these permutations. QTL mapping was conducted using MapQTL 6 (van Ooijen 2011) using Interval Mapping (IM) and subsequent Multiple QTL Model (MQM) mapping.

### Gene & amino acid sequence alignments

Gene lists with annotation information (Bell et al. 2020a) were generated using NovoFinder (Novogene) software. A search was performed within scaffold gene lists to identify candidate genes related to GSL biosynthesis, sugar metabolism, and organic acid metabolism, and raw fasta gene sequences were extracted. Sequences were trimmed to remove non-coding flanking regions and translated using the ExPASy translate tool (Swiss Bioinformatics Resource Portal, Swiss Institute of Bioinformatics, Lausanne, Switzerland). Nucleotide and protein BLAST functions were used to identify similar sequences from related species (https://blast.ncbi.nlm.nih.gov/Blast.cgi, National Centre for Biotechnology Information, Bethesda, MD, USA). *Eruca* reference and alternate SNP sequence alignments were performed using Clustal Omega (European Molecular Biology Laboratory – European Bioinformatics Institute, Hinxton, UK).

### Supplementary Information

**Additional file 1. **Analysis of Variance (ANOVA) summary statistics for glucosinolates, sugars, and organic acids for the *Eruca* mapping population grown in Italy and the UK.

**Additional file 2. **Ranking of *Eruca* mapping population lines for phytochemical composition across the Italy and UK field trials.

**Additional file 3. ***Eruca* linkage map genome scaffolds and gene annotations.

**Additional file 4. **Analysis of Variance (ANOVA) summary of genotype x environment effects on *Eruca* phytochemical concentrations between the two field trial locations (Italy and UK).

**Additional file 5.** Gene sequence BLAST results and associated scores and similarity data.

**Additional file 6.** Protein sequence BLAST results and associated scores and similarity data.

**Additional file 7. ***Eruca* protein sequence alignments with related species for genes identified underlying metabolite QTL.

**Additional file 8. ***Eruca* reference sequence and alternate sequence alignments highlighting the locations of SNPs and changes to the amino acid protein coding sequence.

**Additional file 9. **Protein-protein-interaction networks of *Arabidopsis thaliana* highlighting known links between MYB51 and MYC2 (a), and JAZ5 (also known as TIFY 11A) and MYC2.

**Additional file 10.** KASP marker primer sequences.

## Data Availability

The datasets supporting the conclusions of this article are included within the article (and its additional files). Raw *Eruca* reference sequence and annotation data are available via the European Nucleotide Archive (project PRJEB50993, accession number GCA_932364175). Additional *Eruca* genome, transcriptome, and annotation information is available from LB upon request.

## Abbreviations

4HGB: 4-hydroxyglucobrassicin
4MGB: 4-methoxyglucobrassicin
ANOVA: Analysis Of Variance
ATP: Adenosine Triphosphate
bHLH: Basic Helix-loop-heelix
BLAST: Basic Local Alignment Search Tool
CCOAOMT: Caffeoyl-CoA O-methyltransferase
cM: Centimorgan
DAD: Diode Array Detector
DBD: DNA-binding Domain
DGTB: Diglucothiobeinin
DMB: Dimeric 4-mercaptobutyl glucosinolate
DNA: Deoxyribose Nucleic Acid
F: Self fertilized
GAL: Glucoalyssin
GBC: Glucobrassicin
GER: Glucoerucin
GHP: Glucosinolate Hydrolysis Product
GRA: Glucoraphanin
GRM: Glucorucolamine
GSL: Glucosinolate
GSV: Glucosativin
GxE: Genotype x Environment
HPLC: High Performance Liquid Chromatography
HSD: Honestly Significant Difference
IGMT: Indole Glucosinolate Methyl Transferase
IGV: Integrated Genomics Viewer
JA: Jasmonic Acid
JAZ: Jasmonate ZIM domain-containing
KASP: Kompetitive Allele Specific PCR
ITC: Isothiocyanate
LC-MS: Liquid Chromatography Mass Spectrometry
LG: Linkage Group
LOD: Logarithm Of the Odds
MIM: MYC Interaction Motif
NCBI: National Center for Biotechnology Information
NGB: Neoglucobrassicin
PC: Principal Component
PCA: Principal Component Analysis
PCR: Polymerase Chain Reaction
PRO: Progoitrin
PVDF: Polyvinylidene Fluoride
RI: Refractive Index
RIL: Recombinant Inbred Line
QTL: Quantitative Trait Loci
SF: Sulforaphane
SMART: Simple Modular Architecture Research Tool
SNP: Single Nucleotide Polymorphism
TCA: Tricarboxylic Acid
TSB: Tryptophan Synthase Beta chain
UK: United Kingdom
