# Supplementary material for: Quantitative trait loci analysis of glucosinolate, sugar, and organic acid concentrations in Eruca vesicaria subsp. sativa
Source: Mol Hortic. 2022 Oct 10;2:23. doi: 10.1186/s43897-022-00044-x (PMC10515263; doi:10.1186/s43897-022-00044-x)
Supplement: Supplementary file 4 — Additional file 4. Analysis of Variance (ANOVA) summary of genotype x environment effects on Eruca phytochemical concentrations between the two field trial locations (Italy and UK). [file 43897_2022_44_MOESM4_ESM.docx]

| Levels of significance for genotype x environment ANOVA with *post hoc* Tukey HSD test. | | | |
| --- | --- | --- | --- |
| **Compound** | ***P*-value** | | |
|  | **Genotype** | **Environment** | **GxE** |
| *Glucosinolates* |  |  |  |
| Glucoraphanin | **<0.0001** | **<0.0001** | **<0.0001** |
| Progoitrin | **0.001** | **<0.0001** | 0.203 |
| Glucoalyssin | **<0.0001** | **<0.0001** | **0.013** |
| Diglucothiobeinin | 0.128 | **<0.0001** | 0.730 |
| Glucosativin | 0.070 | **<0.0001** | 0.826 |
| 4-hydroxyglucobrassicin | **<0.0001** | **<0.0001** | 0.432 |
| Glucoerucin | **<0.0001** | **<0.0001** | 0.120 |
| Dimeric-4-mercaptobutyl | **<0.0001** | **<0.0001** | **0.005** |
| 4-methoxyglucobrassicin | **<0.0001** | **<0.0001** | 0.119 |
| Neoglucobrassicin | **<0.0001** | **<0.0001** | **<0.0001** |
| Total GSLs | **<0.0001** | **0.014** | **0.004** |
| *Sugars* |  |  |  |
| Sucrose | 0.093 | **<0.0001** | **0.006** |
| Glucose | **0.011** | **<0.0001** | 0.059 |
| Galactose | **0.021** | **<0.0001** | 0.261 |
| Fructose | 0.637 | **<0.0001** | 0.988 |
| Total sugars | 0.107 | **<0.0001** | 0.409 |
| *Organic acids* |  |  |  |
| Citric | **<0.0001** | **0.004** | 0.134 |
| Malic | **<0.0001** | **<0.0001** | **0.002** |
| Succinic | 0.061 | 0.431 | 0.101 |
| Total acids | **0.000** | **<0.0001** | 0.062 |
