# Supplementary material for: Quantitative trait loci analysis of glucosinolate, sugar, and organic acid concentrations in Eruca vesicaria subsp. sativa
Source: Mol Hortic. 2022 Oct 10;2:23. doi: 10.1186/s43897-022-00044-x (PMC10515263; doi:10.1186/s43897-022-00044-x)
Supplement: Supplementary file 7 — Additional file 7. Eruca protein sequence alignments with related species for genes identified underlying metabolite QTL. [file 43897_2022_44_MOESM7_ESM.docx]

Key:

**Bold** denotes *Eruca sativa* sequence

Colours denote amino acid differences between *Arabidopsis thaliana* (At), *Arabidopsis lyrata* (Al), *Eutrema salsugineum* (Esal), *Raphanus sativus* (Rs), *Eruca sativa* (Es), *Brassica napus* (Bn), *Brassica rapa* (Br) and *Brassica oleracea* (Bo).

**IGMT1**

AtIGMT1 MGYLFQETLSSNPKTPIVVDDDNELGLMAVRLANAAAFPMVLKAALELGVFDTLYAAASR 60

**EsIGMT1a** **MGFPYEETLSSNPKTQTIVDDDNELGLMAVRLANAAAFPMVLKASLELGVFDTLYAEAAR** 60

RsIGMT2 MGFPFEETLSSNPKTQTVVDDDNELGLMAVRLANAAAFPMVLKASLELGVFDTLHAEAAR 60

BoCCOAOMT MGFPFEETLSSNLKTQTVIDDDNELGLMAVRLANAAAFPMVLKASLELGVFDTLYAEAAR 60

BrIGMT4 MGFPFEETLSSNFKTQTVIDDDNELGLMAVRLANAAAFPMVLKASLELGVFDTLYAEAAR 60

BnIGMT4 MGFPFEETLSSNFKTQTVIDDDNELGLMAVRLANAAAFPMVLKASLELGVFDTLYAEAAR 60

**: ::****** ** ::*************************:*********:* *:*

AtIGMT1 TDSFLSPYEIASKLPTTPRNPEAPVLLDRMLRLLASYSMVKCGKALSGKGERVYRAEPIC 120

**EsIGMT1a** **SDTFLSPSEIASRLPTTPRNPEAPVLLDRMLRLLASYSMVKCDKA--GKEERAYRAEPIC** 118

RsIGMT2 TDAFLSPSEIASRLPTTPRNPEAPVLLDRMLRLLASYSMVKCDKA--GKGERTYRAEPIC 118

BoCCOAOMT TDAFLSPSEIASRLPTTPRNPEAPVLLDRMLRLLASYSMVKCDKF--GKGERVYRAEPIC 118

BrIGMT4 TDAFLSPSEIASRLPTTPRNPEAPVLLDRMLRLLASYSMVKCDKV--GKGERVYRAEPIC 118

BnIGMT4 TDAFLSPSEIASRLPTTPRNPEAPVLLDRMLRLLASYSMVKCDKV--GKGERVYRAEPIC 118

:*:**** ****:*****************************.* ** **.*******

AtIGMT1 RFFLKDNIQDIGSLASQVIVNFDSVFLNTWAQLKDVVLEGGDAFGRAHGGMKLFDYMGTD 180

**EsIGMT1a** **RFFLKDNIQDIGSLASQVIVNFDSVFLNTWAQLKDVVLEGGDAFGRAHGGMKLFDYMGTD** 178

RsIGMT2 RFFLKDNIQDIGSLASQVIVNFDSVFLNTWAQLKDVVLEGGDAFGRAHGGMKLFDYMETD 178

BoCCOAOMT RFFLKDNIQDIGSLASQVIVNFDSVFLNTWAQLKDVVLEGGDAFDRAHGGMKLFDYMGTD 178

BrIGMT4 RFFLKDNIQDIGSLASQVIVNFDSVFLNTWAQLKDVVLEGGDAFGRAHGGMKLFDYMGTD 178

BnIGMT4 RFFLKDNIQDIGSLASQVIVNFDSVFLNTWAQLKDVVLEGGDAFGRAHGGMKLFDYMGTD 178

********************************************.************ **

AtIGMT1 ERFSKLFNQTGFTIAVVKKALEVYEGFKGVKVLVDVGGGVGNTLGVVTSKYPNIKGINFD 240

**EsIGMT1a** **ERFSKLFNQTGFTIAVVKKALEVYQGFKDVDVLVDVGGGVGNTLGVVTSKYPNIKGINFD** 238

RsIGMT2 ERFSKLFNQTGFTIAVVKKALEVYQGFEDVDVLVDVGGGVGNTLGVVTSKYPNIKGINFD 238

BoCCOAOMT ERFSKLFNQTGFTIAVVKKALEVYQGFKDVDVLVDVGGGVGNTLGVVTSKYPNIKGVNFD 238

BrIGMT4 ERFSKLFNQTGFTIAVVKKALEVYQGFKDVDVLVDVGGGVGNTLGVVTSKYPNIKGINFD 238

BnIGMT4 ERFSKLFNQTGFTIAVVKKALEVYQGFKDVDVLVDVGGGVGNTLGVVTSKYPNIKGINFD 238

************************:**:.*.*************************:***

AtIGMT1 LTCALAQAPSYPGVEHVAGDMFVDVPTGDAMILKRILHDWTDEDCVKILKNCWKSLPENG 300

**EsIGMT1a** **LICALAQAPSYPGVEHVAGDMFVDVPKGDAMILKRILHDWTDEDCIKILKNCWKSLPENG** 298

RsIGMT2 LTCALAQAPSYPGVEHVAGDMFVDVPKGDAMILKRILHDWTDEDCVKILKNCWKSLPENG 298

BoCCOAOMT LTCALAQAPSYPGVEHVAGDMFVEVPKGDAMILKRILHDWTDEDCVKILKNCWKSLPENG 298

BrIGMT4 LTCALVQAPTYPGVEHVAGDMFVEVPKGDAMILKRILHDWTDEDCIKILKNCWKSLPENG 298

BnIGMT4 LTCALAQAPTYPGVEHVAGDMFVEVPKGDAMILKRILHDWTDEDCIKILKNCWKSLPENG 298

* ***.***:*************:**.******************:**************

AtIGMT1 KVVVIELVTPDEAENGDINANIAFDMDMLMFTQCSGGKERSRAEFEALAAASGFTHCKFV 360

**EsIGMT1a** **KVVVIELVTPDDAENGDINANIAFDMDMLMFTQCSGGKERSRAEFEALAVASGFTQCKFV** 358

RsIGMT2 KVVVIELVTPDDAENGDINANIAFDMDMLMFTQCSGGKERSRAEFEALAIASGFTHCKFV 358

BoCCOAOMT KVVVIELVTPDDADNGDINANIAFDMDMLMFTQCSGGKERSRAEFEALAIASGFTHCKFV 358

BrIGMT4 KVVVIELVTPDDAENGDINANIAFDMDMLMFTQCSGGKERSRAEFEALAVASGFTNCKFV 358

BnIGMT4 KVVVIELVTPDDAENGDINANIAFDMDMLMFTQCSGGKERSRAEFEALAVASGFTNCKFV 358

***********:*:*********************************** *****:****

AtIGMT1 CQAYHCWIIEFCK--- 373

**EsIGMT1a** **CQAYHCWIIEFCKENV** 374

RsIGMT2 CQAYHCWIIEFC---- 370

BoCCOAOMT CQAYHCWIIEFCKENV 374

BrIGMT4 CQAYHCWIIEFCKENV 374

BnIGMT4 CQAYHCWIIEFCKENV 374

************

**IGMT4**

AtIGMT4 MGYLLEETLSSNSKTPIVIDDDNELGLMAVRLANAAAFPMVLKAALELGVFDTLYAEASR 60

BrIGMT4 MGILNEETLSSNPKSQVVIDDDNELGLMAVRLANAAAFPMVLKAALELGVFDTLYAA--- 57

BnIGMT4 MGILNEETLSSNPKSQVVIDDDNELGLMAVRLANAAAFPMVLKAALELGVFDTLYAA--- 57

BoOMT1 MGILNEETLSSNPKSQVVIDDDNELGLMAVRLANAAAFPMVLKAALELGVFDTLYAA--- 57

**EsIGMT4a** **MGILIEETLSSNTKSQIVIDDDNELGLMAVRLANAAAFPMVLKAALELGVFDTLYAA---** 57

RsIGMT4 MGILIEETLNSNANSQIVIDDDNELGLMAVRLANAAAFPMVLKAALELGVFDTLYAA--- 57

** * ****.** :: :***************************************

AtIGMT4 SDSFLSPSEIASKLPTTPRNPEAPVLLDRMLRLLASYSVVKCGKVSEGKGERVYRAEPIC 120

BrIGMT4 -SVFLSPSEIASRLPTTPRNPEAPALLDRMLRLLASYSVVKCGTVQAEKDQRVYKAEPIC 116

BnIGMT4 -SVFLSPSEIASRLPTTPRNPEAPALLDRMLRLLASYSVVKCGTVQAEKDQRVYKAEPIC 116

BoOMT1 -SVFLSPSEIASRLPTTPRNPEAPALLDRMLRLLASYSMVKCGTVQAKKDQRVYKAEPIC 116

**EsIGMT4a** **-SVFLSPSEIASRLPTTPRNPEAPALLDRMLRLLASYSMVKCGTVQAGKGQRVYKAEPIC** 116

RsIGMT4 -SVFLSPSEIASRLPTTPRNPEAPALLDRMLRLLASYSMVKCGTVQAGKGQRVYKAEPIC 116

. *********:***********.*************:****.*. *.:***:*****

AtIGMT4 RFFLKDNIQDIGSLASQVIVNFDSVFLNTWAQLKDVVLEGGDAFGRAHGGMKLFDYMGTD 180

BrIGMT4 RFFLKNNIQDIGSLASQVIVNLDSVFLNTWAQLKDVVLEGGDAFGRAHGGMKLFDYMGTD 176

BnIGMT4 RFFLKNNIQDIGSLASQVIVNSDSVFLNTWAQLKDVVLKGGDAFGRAHGGMKLFDYMGTD 176

BoOMT1 RFFLKNNIQDIGSLASQVIVNSDSVFLNTWAQLKDVVLKGGDAFGRAHGGMKLFDYMGTD 176

**EsIGMT4a** **RFFLKNNIQDIGSLASQVIVNFDSVFLNTWAQLKDVVLEGGDAFGRAHGGMKLFDYMGTD** 176

RsIGMT4 RFFLKDNIQDIGSLASQVIVNFDSVFLNTWAQLKDVVLEGGDAFGRAHGGMKLFDYMGTD 176

*****:*************** ****************:*********************

AtIGMT4 ERFSKLFNQTGFTIAVVKKALEVYQGFKGVNVLVDVGGGVGNTLGVVASKYPNIKGINFD 240

BrIGMT4 ERFSKLFNQTGFTIAVVKKALEVYQGFKDVDVLVDVGGGVGNTLGVVTSKYPNIKGINFD 236

BnIGMT4 ERFSKLFNQTGFTIAVVKKALEVYQGFKDVDVLVDVGGGVGNTLGVVTSNYPNIKGINFD 236

BoOMT1 ERFSKLFNQTGFTIAVVKKALEVYQGFKDVDVLVDVGGGVGNTLGVVTSNYPNIKGINFD 236

**EsIGMT4a**  **ERFSKLFNQTGFTIAVVKKALEVYQGFKDVNVLVDVGGGVGNTLGVVTSKYPNIKGINFD** 236

RsIGMT4 ERFSKLFNQTGFTIAVVKKALEVYQGFKDVDVLVDVGGGVGNTLGVVTSMYPNIKGINFD 236

****************************.*:****************:* **********

AtIGMT4 LTCALAQAPSYPGVEHVAGDMFVDVPTGDAMILKRILHDWTDEDCVKILKNCWKSLPESG 300

BrIGMT4 LTCALAQAPSYPGVEHVAGDMFVEVPKGDTMILKRILHDWTDEDCVKILKNCWKSLPENG 296

BnIGMT4 LTCALAQAPSYPGVEHVAGDMFVEVPKGDTMILKRILHDWTDEDCIKILKNCRKSLPENG 296

BoOMT1 LTCALAQAPSYPGVEHVAGDMFVEVPKGDTMVLKRILHDWTDEDCIKILKNCWKSLPENG 296

**EsIGMT4a** **LTCALAQAPSYPGVEHVAGDMFVEVPKGDAMILKRILHDWTDEDCVKILKNCWKSLPENG** 296

RsIGMT4 LTCALAQAPSYSGVEHVAGDMFVEVPKGDAMILKRILHDWTDEDCVKILKNCWKSLPENG 296

*********** ***********:**.**:*:*************:****** *****.*

AtIGMT4 KVVVIELVTPDEAENGDINANIAFDMDMLMFTQCSGGKERSRAEFEALAAASGFTHCKFV 360

BrIGMT4 KVVVIELVTPDSAESGDINSNIAFDMDMLMFTQCSGGKERSRAEFEALAMESGFTHCKFV 356

BnIGMT4 KVVVIELVTPDNAESGDINANIAFDMDMLMFTQCSGGKERSRAEFEALAMESGFTHCKFV 356

BoOMT1 KVVVIELVTPDNAESGDINANIAFDMDMLMFTQCSGGKERSRAEFEALAMESGFTHCKFV 356

**EsIGMT4a** **KVVVIELVTPENAESGDINSNIAFDMDMLMFTQCSGGKERSRAEFEALAVESGFTHCKFV** 356

RsIGMT4 KVVVIELVTPDNAESGDINSNIAFDMDMLMFTQCSGGKERSRAEFEALAMESGFSHCKFV 356

**********:.**.****:***************************** ***:*****

AtIGMT4 CQAYHCWIIEFCK--- 373

BrIGMT4 CQAYHCWIIEFCKENV 372

BnIGMT4 CQAYHCWIIEFCKENV 372

BoOMT1 CQAYHCWIIEFCKENV 372

**EsIGMT4a** **CQAYHCWIIEFCKENV** 372

RsIGMT4 CQAYHCWVIEFCKENV 372

*******:*****

**JAZ5 (aka TIFY 11A)**

AtJAZ5 MSSSNENAKAQAPEKSDFTRRCSLLSRYLKEKGSFGNIDLGLYRKPDSSLALPGKFDPPG 60

**EsJAZ5** **-MSRNEDGKAQPPEKFNFTRRCSLLSRYLKEKGSFGNIDLGLVRKPELDLGLPGNYDQQE** 59

RsJAZ5 -MSRNEDGKAQPPEKFNFTRRCSLLSRYLKEKGSFGNIDLGLVRKPE-DLRLPGNSDQQD 58

BoJAZ5 -MSRNEDGKAPPPEKSNFTRRCSLLSRYLKEKGSFGNIDLGLVRKPGPDLGLPGNSDQQE 59

BnJAZ5 -MSRNEDGKAPPPEKSNFTRRCSLLSRYLKEKGSFGNIDLGLVRKPGPDLGLPGNSDQQE 59

BrJAZ5 -MSRNEDGEAPPPEKSNFTRRCSLLSRYLKEKGSFGNIDLGLVRKPGPDLGLPGNSDQQE 59

* **:.:* *** :************************* *** .* ***: *

AtJAZ5 KQNAMHKAGH------SKGEPSTSSGGKVKDVADLSESQPGSSQLTIFFGGKVLVYNEFP 114

**EsJAZ5** **KQNVMHKAKG---------ELSSSSGGKAKATNLSEHPDAASSQLTIFFGGKVLVYNEFP** 110

RsJAZ5 KQNVMHKANSEIKALNVLGEPSSLSGGKAKATNLSEPSDPGSSQLTIFFGGKVLVYNEFP 118

BoJAZ5 KQNVMHKANSELKALNVLGEPSISSGGKAKATNLSEPSEPVSSQLTIFFGGKVLVYNEFP 119

BnJAZ5 KQKVMHKANSELKALNVLGEPSSSFGGKAKATNLSEPSEPVSSQLTIFFGGKVLVYNEFP 119

BrJAZ5 KQNVMHKANSELKALNVLGEPSSSFGGKAKATNLSEPSEPISSQLTIFFGGKVLVYNEFP 119

**:.**** * * ***.* . . : *******************

AtJAZ5 VDKAKEIMEVAKQAKPVTEINIQTPINDENNNNKSSMVLPDLNEPTDNNHLTKEQQQQQE 174

**EsJAZ5** **AEKAKEIIQVAKEAKPETEINTQTQIND--HKNKSNMVLPDLNEPTDFADVN--QQQQQQ** 166

RsJAZ5 SDKAKEIIQVAKQAKPVTEVNVQ-------NNNKSNMVLPDLNEPSDSADVN--QQQ-QQ 168

BoJAZ5 SDKAKEIIQVAKEAKSVTDINIQTQINVQKDHNKSNMVLPDLNEPTDTADVN--QQQ-QQ 176

BnJAZ5 SDKAKEIIQVAKEAKSVTDINIQTQINVQKDHNKSNMVLPDLNEPTDTADVN--QQQ-QQ 176

BrJAZ5 SDKAKEIIQVAKEAKSVTDINIQTQINVQKDHNKSNIVLPDLNEPTDTADVN--QQQ-QQ 176

:*****::***:** *::* * .:***.:********:* .:. *** *:

AtJAZ5 QNQIVERIARRASLHRFFAKRKDRAVARAPYQVNQNAGHHRYPPKPEIVTGQPLEAGQSS 234

**EsJAZ5** **QNQLVERIARRASLHRFFAKRKDRAVARAPYQVNQNAGRHHYPPKPETLPGQQREQGQSS** 226

RsJAZ5 QNQLVERIARRASLHRFFAKRKDRAVARAPYQVNQNAGSHHYPPKPD------------- 215

BoJAZ5 QNQLVERIARRASLHRFFAKRKDRAVARAPYQVNQTGGGHHYPPKPETVPGQQLEQGQSS 236

BnJAZ5 QNQLVERIARRASLHRFFAKRKDRAVARAPYQVNQNGGGHHYPPKPETVPGQQLEQGQSS 236

BrJAZ5 QNQLVERIARRASLHRFFAKRKDRAVARAPYQVNQNGGGHHYPPKPETVPGQQLEQGQSS 236

***:*******************************..* *:*****:

AtJAZ5 QRPPDNAIGQTMAHIKSDGDKDDIMKI-EEGQSSKDLDLRL 274

**EsJAZ5** **QR-PDTAVAQTVSHPKPECAKDMLMEVKGEGQCSKDLELRL** 266

RsJAZ5 ----------------------------------------- 215

BoJAZ5 ---Q------RPAQPKPECDKDMLMEVKEDGQCSKDLELRL 268

BnJAZ5 QP-Q------RPAQPKPECDKDMLME---EGQCSKDLELRL 267

BrJAZ5 QP-Q------RPAQPKPECDKDMLMEVKEEGQCSKDLELRL 270

**MYB51**

AtMYB51 -----------------------------------------------------------M 1

RsMYB51 -----------------------------------------------------------M 1

**EsMYB51b** **MFELSNQKIMCSIVMIICPLSHFVSTLSFSIFFFSLFHISLGNNRFERDQNNHKNQELKM** 60

BnMYB51 -----------------------------------------------------------M 1

BoMYB51 -----------------------------------------------------------M 1

BrMYB51 -----------------------------------------------------------M 1

*

AtMYB51 VRTPCCKAELGLKKGAWTPEEDQKLLSYLNRHGEGGWRTLPEKAGLKRCGKSCRLRWANY 61

RsMYB51 VRTPCCKAELGLKKGAWTPEEDQKLVSYVNRHGEGGWRTLPEKAGLKRCGKSCRLRWANY 61

**EsMYB51b** **VRTPCCKAELGLKKGAWTPEEDQKLVSYVNRHGEGGWRTLPEKAGLKRCGKSCRLRWANY** 120

BnMYB51 VRTPCCKAELGLKKGAWTPEEDQKLVSYVNRHGEGGWRTLPEKAGLKRCGKSCRLRWANY 61

BoMYB51 VRTPCCKAELGLKKGAWTPEEDQKLVSYVNRHGEGGWRTLPEKAGLKRCGKSCRLRWANY 61

BrMYB51 VRTPCCKAELGLKKGAWTPEEDQKLVSYVNCHGEGGWRTLPEKAGLKRCGKSCRLRWANY 61

*************************:**:* *****************************

AtMYB51 LRPDIKRGEFTEDEERSIISLHALHGNKWSAIARGLPGRTDNEIKNYWNTHIKKRLIKKG 121

RsMYB51 LRPDIKRGEFTEDEERSIISLHALHGNKWAAIARGLPGRTDNEIKNHWNTHIKKRLIKKG 121

**EsMYB51b** **LRPDIKRGEFTEDEERSIISLHALHGNKWAAIARGLPGRTDNEIKNHWNTHIKKLLIKKG** 180

BnMYB51 LRPDIKRGEFTEDEECSIISLHALHGNKWAAIARGLPGRTDNEIKNHWNTHIKKRLIKKG 121

BoMYB51 LRPDIKRGEFTEDEECSIISLHALHGNKWAAIARGLPGRTDNEIKNHWNTHIKKRLIKKG 121

BrMYB51 LRPDIKRGEFTEDEERSIISLHALHGNKWAAIARGLPGRTDNEIKNHWNTHIKKRLIKKG 121

*************** *************:****************:******* *****

AtMYB51 IDPVTHKGITSGTD-KSENLPEKQNV--NLTTSDHDLDNDKAKKNNKNFGLSSASFLNKV 178

RsMYB51 VDPVTHKS------VNLPEIPEKQNVIPTIITSDVDLDNEKMKNNNKKPGLSSAKFLNRV 175

**EsMYB51b** **VDPVTHKSLISDKSENLPEIPEKQNVIQEIITSGDNLDKEEVKNDNKKSGLSSARFLNRV** 240

BnMYB51 VDPVTHKSLISDKSENFPEIPEKQNVIQTIITSEDDLDNEKVKNSNKKPVLSSAKFLNRV 181

BoMYB51 VDPVTHKSLISDKSENFPEIPEKQNVIQTIITSEDDLDNEKVKNSNKKPVLSSAKFLNRV 181

BrMYB51 VDPVTHKSLISDKSENLPEIPEKQNVIQTIITSDDDLDNEKVKNNNEKPGLSSAKFLNRV 181

:******. : ::****** : ** :**::: *:.*:: **** ***:*

AtMYB51 ANRFGKRINQSVLSEIIGSGGPLASTSHT-TNTTTTSVSVDSESVKSTSSSFAPTS-NLL 236

RsMYB51 ANRFGKRINQSVLSDIIGSGGPVTTITTSHTAATTTSVTVNSESDKSTSSSFTPAS-SLP 234

**EsMYB51b** **ANRFGKRINQSVLSEIIGSGGPLTSTTTSHTT-TTTSVTINSESDKSISSSFTPTSSDLL** 299

BnMYB51 ANRFGKRINQSVLSEIIGSGGPLTTTTTSHTA-TTTSVTVNSESDKSTSSSFTPT-SDLL 239

BoMYB51 ANRFGKRINQSVLSEIIGSGGPLTTTTTSHTA-TTTSVTVNSESDKSTSSSFTPT-SDLL 239

BrMYB51 ANRFGKRINQSVLSEIIGSGGPLTTTTTSHTA-TTTSVTVNSESDKSTSSSFTPT-SDLL 239

**************:*******::: : : * *****:::*** ** ****:*: .*

AtMYB51 CHGTVATTPVSSNFDVDGNVNLTCSSSTFSDSSVNNPLMYCDNFVGNNNVDDEDTIGFST 296

RsMYB51 CQMTV-------------NGNATSSPSTFSDASVNDHLMHCDN---------EDNIEFSR 272

**EsMYB51b** **CQMTV-------------NGNATSSPSTFSDASVNDSLMYCDN---------EDNLGFSN** 337

BnMYB51 CQMTV-------------NGNATSSPSTFSDASVNDSLMYCDN---------EDNLGFSN 277

BoMYB51 CQMTV-------------NGNATSSPSTFSDASVNDSLMYCDN---------EDNLGFSN 277

BrMYB51 CQMTV-------------NGNATSSPSTFSEASVNDSLMYCDN---------EDNLGFSN 277

*: ** * * *.* ****::***: **:*** **.: **

AtMYB51 FLNDEDFMMLEESCVENTAFMKELTRFLHEDENDVVDVTPVYERQDLFDEIDNYFG 352

RsMYB51 FLNDEDFMMFEESCVDNTEFMKELTRFLEEDVNDDLEVMPVYEHQEKFEEIDNYFA 328

**EsMYB51b** **FLNDEDFMMFGESCVDNTEFMKELTSFLQEDVSDDVQVMPVNEHKDNIEETDNYFA** 393

BnMYB51 FLNDEDFMMLEESCVDNTEFMKELSRFLEEDVNDDVEVMHVYEHQDNIEDIDNYFA 333

BoMYB51 FLNDEDFMMLEESCVDNTEFMKELSRFLEEDVNDDVEVMHVYEHQDNIEDIDNYFA 333

BrMYB51 FLNDEDFMMLEESSVDNTEFMKELSRFLEEDVNDDVEVMPVYEHQDNIEDIDNYFA 333

*********: **.*:** *****: **.** .* ::* * *::: ::: ****.

**TSB**

AtTSB1 MAASGTSATFRASVSSAPSSSSQLTHLKSPFKAVKYTPLPSSRSK--SSSFSVSCTI--- 55

BoTSB1 MFGKEKSQI--ISVSKKKK---NRNMSSTKIFQVRGQPLPRFPVRNHRMINTVVCGFPII 55

**EsTSB-like --------------------------MSSTRTQVRGEPLPRVPARYHRMINFFVCGVPIK** 34

RsTSB1 --------------------------MSSTKIQVRGQPLPMVLTRNHRMINSVVCGVQNK 34

BnTSB1 --------------------------MSSTKIQIRGQPLFKVLTRNHRMINSVVCGVPIK 34

BrTSB1 --------------------------MSSTKIQIRGQPLFKVLTRNHRMINSVVCGVPIK 34

.: :: ** : . * .

AtTSB1 -----------AKDPP-VLMAAGSDPALWQRPDSFGRFGKFGGKYVPETLMHALSELESA 103

BoTSB1 SHHRVSNVLSRTSGPFLGYVPARTDENPFLRGDSNGRFGKFGGKFVPETLMSCLRDLEDE 115

**EsTSB-like THHRVSNVL-RTSGSSLGSVPTRTEESQFLRGDVNGRFGRFGGKFVPETLMSPLRDLEDV** 93

RsTSB1 RHHYVSNVL-RTIDPPLGSVPTRTDDSQFLRGDGNGRFGRFGGKFVPETLISPLKYLEDE 93

BnTSB1 RQHRVSNVL-RTSDPPLGSVPTRTDESQFLRGDGNGRFGRFGGKFVPETLMSPLRDLEDE 93

BrTSB1 RQHRVSNVL-RTSDPPLGSVPTRTDESQFLRGDGNGRFGRFGGKFVPETLMSPLRDLEDE 93

: . : : :: : * * ****:****:*****: * **.

AtTSB1 FYALATDDDFQRELAGILKDYVGRESPLYFAERLTEHYRR---ENGEGPLIYLKREDLNH 160

BoTSB1 FNFVLSDHEFQVEFTAALRDYVGRETPLYFAERLTQHYKNIARTTGDGPEIYLKREDLCH 175

**EsTSB-like FNFVLSDREFQEELTTALREYVGRETPLYFAGRLTEHYKNISQTTGGGPQIYLKREDLSH** 153

RsTSB1 FNFVLSDHEFQEELTTALRDYVGRETPLYFAGRLTEHYKNISQTTGGGPEIYLKREDLSH 153

BnTSB1 FDFVLNDHEFQEELTTALRDYVGRETPLYFAGRLTEHYKNISQTTGGGPEIYLKREDLSH 153

BrTSB1 FDFVLNDHEFQEELTTALRDYVGRETPLYFAGRLTEHYKNISQTTGGGPEIYLKREDLSH 153

* : .* :** *:: *::*****:***** ***:**:. .* ** ******** *

AtTSB1 TGAHKINNAVAQALLAKRLGKKRIIAETGAGQHGVATATVCARFGLECIIYMGAQDMERQ 220

BoTSB1 GGSHKINNALAQAMIARRLGCSRVVAATGSGQHGVATAAACAKLSLECTVFMGTTDIEKQ 235

**EsTSB-like CGSHKINNALGQAMIARRLGCKRVVAATGAGQHGVATAAACAKLSLECTVFMGTTDIEKQ** 213

RsTSB1 CGSHKINNALAQAMIARRLGCSRVVAATGAGQHGVATAAACAKLSLECTVFMGSTDIEKQ 213

BnTSB1 CGSHKINNALGQAMIARRLGCKRVVAATGAGQHGVATAAACAKLSMECTVFMGTTDIEKQ 213

BrTSB1 CGSHKINNALGQAMIARRLGCKRVVAATGAGQHGVATAAACAKLSIECTVFMGTTDIEKH 213

*:******:.**::*:*** .*::* **:********:.**::.:** ::**: *:*::

AtTSB1 ALNVFRMRLLGAEVRGVHSGTATLKDATSEAIRDWVTNVETTHYILGSVAGPHPYPMMVR 280

BoTSB1 SSNVLSMKLLGAQVKSVQ---GTFQDASSEAIRNWVGKLETTYYLPGTVVGPHPSPVMVR 292

**EsTSB-like SSNVLSMKLLGAQVKSVE---GRFKDASSEAIRNWVGNLETTYYLSGTVVGPHPSPLMVR** 270

RsTSB1 SSNVLSMKLLGAQVKSVE---GTFKDASSEAIRNWVGNLETTYYLSGTVVGPHPSPLMVR 270

BnTSB1 SSNVLSMKLLGAQVKSVE---GTFKDASSEAIRNWVGNLETTYYLSGTVVGPHPSPLMVR 270

BrTSB1 SSNVLSMKLLGAQVKSVE---GTFKDASSEAIRNWVGNLETTYYLSGTVVGPHPNPLMVR 270

: **: *:****:*:.*. . ::**:*****:** ::***:*: *:*.**** *:***

AtTSB1 DFHAVIGKETRKQALEKWGGKPDVLVACVGGGSNAMGLFHEFVNDTEVRMIGVEAAGFGL 340

BoTSB1 EFQSVIGKETRRQAKQLWGGKPDVLVACVGSGSNALGLFHEFVRDEDVRLVGVEAAGLGL 352

**EsTSB-like EFQSVIGKETRRQANQLWGGKPDVLLACVGSGSNALGLFHEFLGDEDVRLVGIEAAGLGL** 330

RsTSB1 EFQSVIGKETRRQANQLWGGKPDVLVACIGSGSNALGLFHEFVGDEDVRLVGVEAAGLGL 330

BnTSB1 EFQSVIGKETRRQAKQLWGGKPDVLVACVGSGSNALGLFHEFLGDEDVRLVGVEAAGLGL 330

BrTSB1 EFQSVIGKETRRQAKQLWGGKPDVLVACVGSGSNALGLFHEFLGDEDVRLVGVEAAGLGL 330

:*::*******:** : ********:**:*.****:******: * :**::*:****:**

AtTSB1 DSGKHAATLTKGDVGVLHGAMSYLLQDDDGQIIEPHSISAGLDYPGVGPEHSFFKDMGRA 400

BoTSB1 DSGKHSATLAVGDVGVYHGSMSYLLQDDQGQILRPHSIGVGLEYPGVGPEISFLKESGRA 412

**EsTSB-like DSGKHSATLAVGDVGVYHGSMSYLLQDDQGQIVKPHSVGVGLEYPGVGPEISFLKETGRA** 390

RsTSB1 DSGKHSATLAVGDVGVYHGSMSYLLQDDQGQILEPHSVGVGLEYPGVGPEISFLKETGRA 390

BnTSB1 DSGKHSATLAVGDVGVYHGSMSYLLQDDQGQILKPHSIGVGLEYPGVGPEISFLKESGRA 390

BrTSB1 DSGKHSATLAVGDVGVYHGSMSYLLQDDQGQILKPHSIGVGLEYPGVGPEISFLKESGRA 390

*****:***: ***** **:********:***:.***:..**:******* **:*: ***

AtTSB1 EYYSITDEEALEAFKRVSRLEGIIPALETSHALAYLEKLCPTLSDGTRVVLNFSGRGDKD 460

BoTSB1 EFCTATDQEAIQACMLLSRLEGIIPALETSHALAILEKLVPTLRDGAKVVVNCSGRGDKD 472

**EsTSB-like EFYTATDQEAIQACMLLSRLEGIIPALEPSHALAFLDKLVPTLPDGAKVVVNCSGRGDKD** 450

RsTSB1 EFYTATDQEAIQACMLLSRLEGIIPALEASHALAFLDKLVPTLRDGTKVVVNCSGRGDKD 450

BnTSB1 EFYTATDQEAVQACMLLSRLEGIIPALEASHALAFLDKLVPTLRDGAKVVVNCSGRGDKD 450

BrTSB1 EFYTATDQEAVQACMLLSRLEGIIPALEASHALAFLDKLVPTLRDGAKVVVNCSGRGDKD 450

*: : **:**::* :*********** ***** *:** *** **::**:* *******

AtTSB1 VQTVAKYLDV---- 470

BoTSB1 INTLIQRGMPSSLC 486

**EsTSB-like LNTLIQRGVLSSLC** 464

RsTSB1 LDILIQRGMPSSLR 464

BnTSB1 LDTLIQRGMPSSLC 464

BrTSB1 LDTLIQRGMPSSLC 464

:: : :
