# Supplementary material for: Quantitative trait loci analysis of glucosinolate, sugar, and organic acid concentrations in Eruca vesicaria subsp. sativa
Source: Mol Hortic. 2022 Oct 10;2:23. doi: 10.1186/s43897-022-00044-x (PMC10515263; doi:10.1186/s43897-022-00044-x)
Supplement: Supplementary file 8 — Additional file 8. Eruca reference sequence and alternate sequence alignments highlighting the locations of SNPs and changes to the amino acid protein coding sequence. [file 43897_2022_44_MOESM8_ESM.docx]

**IGMT1**

**DNA alignment**

Ref ATGGGATTCCCTTATGAAGAAACCTTGAGCTCTAACCCTAAAACCCAAACTATTGTTGAT 60

Alt ATGGGATTCCCTTATGAAGAAACCTTGAGCTCTAACCCTAAAACCCAAACTGTTGTTGAT 60

*************************************************** ********

Ref GATGATAATGAGTTGGGTTTGATGGCTGTGAGACTAGCCAATGCCGCAGCCTTTCCCATG 120

Alt GATGATAATGAGTTGGGTTTGATGGCTGTGAGACTAGCCAATGCCGCTGCCTTTCCCATG 120

*********************************************** ************

Ref GTTCTCAAAGCCTCCCTCGAGCTCGGTGTCTTTGACACTCTTTACGCCGAAGCTGCTCGT 180

Alt GTTCTCAAAGCCTCCCTCGAGCTCGGTGTCTTTGACACTCTTTACGCCGAAGCTGCTCGT 180

************************************************************

Ref TCCGACACCTTCCTCTCACCATCTGAGATAGCGAGCAGGCTACCAACTACACCACGTAAC 240

Alt TCCGACACCTTCCTCTCACCATCTGAGATAGCGAGCAGGCTACCAACTACACCACGTAAC 240

************************************************************

Ref CCTGAGGCTCCGGTTTTGTTGGACCGGATGCTTCGTCTACTCGCTAGCTACTCCATGGTC 300

Alt CCTGAGGCTCCGGTTTTGTTGGACCGGATGCTTCGTCTACTCGCTAGCTACTCCATGGTC 300

************************************************************

Ref AAATGCGATAAGGCTGGGAAGGAAGAGAGAGCCTATAGAGCTGAGCCAATTTGTAGATTT 360

Alt AAATGCGATAAGGCTGGGAAGGAAGAGAGAGCCTATAGAGCTGAGCCAATTTGTAGATTT 360

************************************************************

Ref TTCTTGAAGGATAATATTCAAGATATAGGTTCCCTTGCTTCTCAAGTCATTGTCAATTTT 420

Alt TTCTTGAAGGATAATATTCAAGATATAGGTTCCCTTGCTTCTCAAGTCATTGTCAATTTT 420

************************************************************

Ref GACAGTGTCTTCCTCAATACATGGGCACAACTGAAAGATGTGGTACTTGAAGGAGGAGAC 480

Alt GACAGCGTCTTCCTCAATACATGGGCACAACTGAAAGATGTGGTACTTGAAGGAGGAGAT 480

***** *****************************************************

Ref GCATTTGGCCGTGCACATGGTGGCATGAAACTCTTTGACTATATGGGAACTGATGAGAGA 540

Alt GCATTTGGCCGTGCACATGGTGGCATGAAACTCTTTGACTATATGGGAACTGATGAGAGA 540

************************************************************

Ref TTCAGCAAGCTCTTTAACCAGACCGGGTTCACCATCGCTGTGGTGAAGAAGGCTCTTGAA 600

Alt TTCAGCAAGCTCTTTAACCAGACCGGGTTCACCATTGCTGTGGTGAAGAAGGCTCTTGAA 600

*********************************** ************************

Ref GTTTATCAAGGCTTTAAAGATGTGGATGTGTTGGTTGATGTTGGAGGTGGAGTTGGAAAC 660

Alt GTTTATCAAGGCTTTAAAGATGTGGATGTGTTGGTTGATGTTGGAGGTGGAGTTGGAAAC 660

************************************************************

Ref ACTCTTGGTGTTGTTACTTCTAAGTATCCTAATATTAAGGGTATTAACTTTGATCTGATC 720

Alt ACTCTTGGTGTTGTTACTTCTAAGTATCCTAATATTAAGGGTATTAACTTTGATCTGATC 720

************************************************************

Ref TGTGCCTTGGCACAAGCACCTTCTTACCCTGGTGTGGAACATGTAGCGGGAGATATGTTT 780

Alt TGTGCCTTGGCACAAGCACCTTCTTACCCTGGTGTGGAACATGTAGCGGGAGATATGTTT 780

************************************************************

Ref GTGGATGTTCCAAAGGGAGATGCCATGATCTTGAAACGTATACTTCATGATTGGACCGAC 840

Alt GTGGATGTTCCAAAGGGAGATGCCATGATCTTGAAACGTATACTTCATGATTGGACCGAC 840

************************************************************

Ref GAAGACTGCATTAAGATTCTCAAGAACTGTTGGAAATCACTACCAGAGAACGGTAAAGTT 900

Alt GAAGACTGCATTAAGATTCTCAAGAACTGTTGGAAATCACTACCAGAGAACGGTAAAGTT 900

************************************************************

Ref GTTGTCATTGAACTAGTCACTCCTGATGATGCAGAGAATGGGGATATCAACGCGAACATT 960

Alt GTTGTCATTGAACTAGTCACTCCTGATGATGCAGAGAATGGGGATATCAACGCGAACATT 960

************************************************************

Ref GCATTTGATATGGATATGTTGATGTTCACCCAATGTTCTGGTGGAAAAGAAAGGTCACGA 1020

Alt GCATTTGATATGGATATGTTGATGTTCACCCAATGTTCTGGTGGAAAAGAAAGGTCACGA 1020

************************************************************

Ref GCCGAGTTTGAAGCTTTGGCTGTAGCTTCTGGCTTTACCCAATGCAAATTCGTTTGCCAG 1080

Alt GCCGAGTTTGAAGCTTTGGCTGTAGCTTCTGGCTTTACCCAATGCAAATTCGTTTGCCAG 1080

************************************************************

Ref GCTTATCACTGCTGGATTATCGAGTTTTGTAAAGAAAATGTG 1122

Alt GCTTATCACTGCTGGATTATCGAGTTTTGTAAAGAAAATGTG 1122

******************************************

**Protein alignment**

Ref MGFPYEETLSSNPKTQTIVDDDNELGLMAVRLANAAAFPMVLKASLELGVFDTLYAEAAR 60

Alt MGFPYEETLSSNPKTQTVVDDDNELGLMAVRLANAAAFPMVLKASLELGVFDTLYAEAAR 60

*****************:******************************************

Ref SDTFLSPSEIASRLPTTPRNPEAPVLLDRMLRLLASYSMVKCDKAGKEERAYRAEPICRF 120

Alt SDTFLSPSEIASRLPTTPRNPEAPVLLDRMLRLLASYSMVKCDKAGKEERAYRAEPICRF 120

************************************************************

Ref FLKDNIQDIGSLASQVIVNFDSVFLNTWAQLKDVVLEGGDAFGRAHGGMKLFDYMGTDER 180

Alt FLKDNIQDIGSLASQVIVNFDSVFLNTWAQLKDVVLEGGDAFGRAHGGMKLFDYMGTDER 180

************************************************************

Ref FSKLFNQTGFTIAVVKKALEVYQGFKDVDVLVDVGGGVGNTLGVVTSKYPNIKGINFDLI 240

Alt FSKLFNQTGFTIAVVKKALEVYQGFKDVDVLVDVGGGVGNTLGVVTSKYPNIKGINFDLI 240

************************************************************

Ref CALAQAPSYPGVEHVAGDMFVDVPKGDAMILKRILHDWTDEDCIKILKNCWKSLPENGKV 300

Alt CALAQAPSYPGVEHVAGDMFVDVPKGDAMILKRILHDWTDEDCIKILKNCWKSLPENGKV 300

************************************************************

Ref VVIELVTPDDAENGDINANIAFDMDMLMFTQCSGGKERSRAEFEALAVASGFTQCKFVCQ 360

Alt VVIELVTPDDAENGDINANIAFDMDMLMFTQCSGGKERSRAEFEALAVASGFTQCKFVCQ 360

************************************************************

Ref AYHCWIIEFCKENV 374

Alt AYHCWIIEFCKENV 374

**************

Residue 18 – isoleucine to valine

Functional domains based on Arabidopsis sequence

**IGMT4**

**DNA alignment**

Ref ATGGGAATCCTTATTGAAGAAACCTTAAGCTCTAACACCAAAAGCCAAATTGTTATTGAT 60

Alt ATGGGAATCCTTATTGAAGAAACCTTAAGCTCTAACACCAAAAGCCAAATTGTTATTGAT 60

************************************************************

Ref GATGATAATGAGTTGGGTTTAATGGCCGTGAGACTAGCCAATGCTGCTGCCTTTCCTATG 120

Alt GATGATAATGAGTTGGGTTTAATGGCCGTGAGACTAGCCAATGCTGCTGCCTTTCCTATG 120

************************************************************

Ref GTTCTCAAAGCTGCCCTTGAGCTCGGTGTCTTTGACACTCTCTACGCCGCCTCTGTCTTC 180

Alt GTTCTCAAAGCTGCCCTTGAGCTCGGTGTCTTTGACACTCTCTACGCCGCCTCTGTCTTC 180

************************************************************

Ref CTCTCACCTTCCGAGATAGCAAGTAGGCTACCAACTACACCTCGTAACCCTGAGGCTCCG 240

Alt CTCTCACCTTCCGAGATAGCAAGTAGGCTACCAACTACACCTCGTAACCCTGAGGCTCCG 240

************************************************************

Ref GCTTTGTTGGACAGGATGCTTCGTCTACTAGCTAGCTACTCCATGGTCAAGTGTGGTACG 300

Alt GCTTTGTTGGACAGGATGCTTCGTCTACTAGCTAGCTACTCCATGGTCAAGTGTGGTACG 300

************************************************************

Ref GTCCAAGCTGGAAAGGGCCAGAGAGTTTACAAAGCCGAGCCAATATGCAGGTTTTTCTTG 360

Alt GTCCAAGCTGGAAAGGGCCAGAGAGTTTACAAAGCCGAGCCAATATGCAGGTTTTTCTTG 360

************************************************************

Ref AAAAATAACATTCAAGATATTGGATCCTTAGCTTCTCAAGTCATTGTCAATTTCGACAGT 420

Alt AAAAATAACATTCAAGATATTGGATCCTTAGCTTCTCAAGTCATTGTCAATTTCGACAGT 420

************************************************************

Ref GTCTTCCTCAACACCTGGGCACAACTGAAAGATGTTGTACTTGAAGGAGGAGATGCATTT 480

Alt GTCTTCCTCAACACCTGGGCACAACTGAAAGATGTTGTACTTGAAGGAGGAGATGCATTT 480

************************************************************

Ref GGCCGTGCACATGGTGGCATGAAACTCTTCGACTATATGGGAACTGATGAGAGATTCAGC 540

Alt GGCCGTGCACATGGTGGCATGAAACTCTTCGACTATATGGGAACTGATGAGAGATTCAGC 540

************************************************************

Ref AAGCTCTTTAACCAGACAGGATTCACCATTGCTGTCGTGAAGAAGGCTCTTGAAGTTTAC 600

Alt AAGCTCTTTAACCAGACAGGATTCACCATTGCTGTCGTGAAGAAGGCTCTTGAAGTTTAC 600

************************************************************

Ref CAAGGCTTCAAAGATGTGAATGTGTTAGTTGATGTAGGAGGAGGAGTTGGAAATACCCTT 660

Alt CAAGGCTTCAAAGATGTGAATGTGTTAGTTGATGTAGGAGGAGGAGTTGGAAACACCCTT 660

***************************************************** ******

Ref GGTGTTGTTACCTCTAAGTATCCTAATATTAAGGGTATAAATTTTGATCTTACTTGTGCC 720

Alt GGTGTAGTTACCTCTAAGTATCCTAATATTAAGGGTATAAATTTTGATCTTACTTGTGCC 720

***** ******************************************************

Ref TTGGCACAAGCACCTTCTTACCCTGGGGTGGAACATGTGGCCGGAGATATGTTTGTGGAA 780

Alt TTGGCACAAGCACCTTCTTACCCTGGGGTGGAACATGTGGCCGGAGATATGTTTGTGGAA 780

************************************************************

Ref GTTCCAAAGGGAGATGCCATGATCTTGAAACGTATACTTCATGATTGGACTGACGAAGAC 840

Alt GTTCCAAAGGGAGATGCCATGATCTTGAAACGTATACTTCATGATTGGACTGACGAAGAC 840

************************************************************

Ref TGCGTAAAAATTCTTAAAAACTGTTGGAAATCACTTCCTGAAAATGGCAAAGTGGTCGTG 900

Alt TGCGTAAAGATTCTTAAAAACTGTTGGAAATCACTTCCTGAAAATGGCAAAGTGGTCGTG 900

******** ***************************************************

Ref ATAGAGCTAGTGACTCCTGAAAATGCTGAGAGTGGAGACATAAACTCGAACATTGCATTT 960

Alt ATAGAGCTAGTGACTCCTGATAATGCTGAGAGTGGAGACATAAACTCGAACATTGCATTT 960

******************** ***************************************

Ref GATATGGACATGTTGATGTTCACACAATGTTCGGGTGGAAAAGAGAGGTCTCGAGCTGAG 1020

Alt GATATGGACATGTTGATGTTCACACAATGTTCCGGTGGAAAAGAGAGGTCTCGAGCTGAG 1020

******************************** ***************************

Ref TTTGAAGCTTTAGCGGTGGAATCAGGCTTCACCCATTGCAAATTCGTTTGTCAGGCTTAT 1080

Alt TTTGAAGCTTTAGCGGTGGAATCAGGCTTCACCCATTGCAAATTCGTTTGTCAGGCTTAT 1080

************************************************************

Ref CACTGTTGGATTATTGAGTTCTGTAAAGAAAATGTT 1116

Alt CACTGCTGGATTATTGAGTTCTGTAAAGAAAATGTT 1116

***** ******************************

**Protein alignment**

Ref MGILIEETLSSNTKSQIVIDDDNELGLMAVRLANAAAFPMVLKAALELGVFDTLYAASVF 60

Alt MGILIEETLSSNTKSQIVIDDDNELGLMAVRLANAAAFPMVLKAALELGVFDTLYAASVF 60

************************************************************

Ref LSPSEIASRLPTTPRNPEAPALLDRMLRLLASYSMVKCGTVQAGKGQRVYKAEPICRFFL 120

Alt LSPSEIASRLPTTPRNPEAPALLDRMLRLLASYSMVKCGTVQAGKGQRVYKAEPICRFFL 120

************************************************************

Ref KNNIQDIGSLASQVIVNFDSVFLNTWAQLKDVVLEGGDAFGRAHGGMKLFDYMGTDERFS 180

Alt KNNIQDIGSLASQVIVNFDSVFLNTWAQLKDVVLEGGDAFGRAHGGMKLFDYMGTDERFS 180

************************************************************

Ref KLFNQTGFTIAVVKKALEVYQGFKDVNVLVDVGGGVGNTLGVVTSKYPNIKGINFDLTCA 240

Alt KLFNQTGFTIAVVKKALEVYQGFKDVNVLVDVGGGVGNTLGVVTSKYPNIKGINFDLTCA 240

************************************************************

Ref LAQAPSYPGVEHVAGDMFVEVPKGDAMILKRILHDWTDEDCVKILKNCWKSLPENGKVVV 300

Alt LAQAPSYPGVEHVAGDMFVEVPKGDAMILKRILHDWTDEDCVKILKNCWKSLPENGKVVV 300

************************************************************

Ref IELVTPENAESGDINSNIAFDMDMLMFTQCSGGKERSRAEFEALAVESGFTHCKFVCQAY 360

Alt IELVTPDNAESGDINSNIAFDMDMLMFTQCSGGKERSRAEFEALAVESGFTHCKFVCQAY 360

******:*****************************************************

Ref HCWIIEFCKENV 372

Alt HCWIIEFCKENV 372

************

Residue 307 – glutamic acid to aspartic acid

Functional domains based on Arabidopsis sequence

**MYB51**

**DNA alignment**

Ref ATGTTTGAACTCTCTAATCAAAAGATCATGTGCTCAATCGTTATGATCATATGTCCCCTT 60

Alt ATGTTTGAACTCTCTAATCAAAAGATCATGTGCTCAATCGTTATGATCATATGTCCCCTT 60

************************************************************

Ref TCACACTTTGTTTCAACTCTCAGTTTTTCAATTTTTTTTTTCTCTTTGTTCCATATATCA 120

Alt TCACACTTTGTTTCAACTCTCAGTTTTTCAATTTTTTTTTTCTCTTTGTTCCATATATCA 120

************************************************************

Ref CTTGGGAACAACCGCTTCGAACGCGATCAAAATAATCACAAAAATCAAGAACTCAAGATG 180

Alt CTTGGGAACAACCGCTTCGAACGCGATCAAAATAATCACAAAAATCAAGAACTCAAGATG 180

************************************************************

Ref GTGCGAACACCATGTTGCAAAGCTGAACTAGGGTTGAAGAAGGGAGCATGGACTCCAGAG 240

Alt GTGCGAACACCATGTTGCAAAGCTGAGCTAGGGTTGAAGAAGGGAGCATGGACTCCCGAG 240

************************** ***************************** ***

Ref GAAGATCAGAAGCTTGTCTCCTACGTCAACCGTCACGGTGAAGGTGGATGGCGAACTCTC 300

Alt GAAGATCAGAAGCTTGTCTCCTACGTCAACCGTCACGGTGAAGGTGGATGGCGAACTCTC 300

************************************************************

Ref CCCGAAAAAGCTGGACTCAAGAGATGTGGCAAAAGCTGCAGACTGAGATGGGCCAATTAT 360

Alt CCCGAAAAAGCTGGACTCAAGAGATGTGGCAAAAGCTGCAGACTGAGATGGGCCAATTAT 360

************************************************************

Ref CTAAGACCTGACATCAAAAGAGGAGAGTTCACTGAAGATGAAGAACGTTCTATCATCTCT 420

Alt CTAAGACCTGACATCAAAAGAGGAGAGTTCACTGAAGATGAAGAACGTTCTATCATCTCT 420

************************************************************

Ref CTCCATGCCCTTCATGGCAACAAATGGGCTGCAATAGCTCGTGGATTACCAGGAAGAACC 480

Alt CTCCATGCCCTTCATGGCAACAAATGGGCTGCAATAGCGCGTGGATTACCAGGAAGAACC 480

************************************** *********************

Ref GATAACGAAATCAAGAACCACTGGAACACTCATATCAAAAAACTTTTGATCAAAAAAGGT 540

Alt GATAACGAAATCAAGAACCACTGGAACACTCATATCAAAAAACTTTTGATCAAAAAAGGT 540

************************************************************

Ref GTCGATCCGGTTACACACAAGAGCCTGATCTCCGACAAATCAGAAAACCTCCCGGAGATT 600

Alt GTCGATCCGGTTACACACAAGAGCTTGATCTCCGACAAATCAGAAAACCTCCCGGAGATT 600

************************ ***********************************

Ref CCAGAGAAACAAAACGTTATTCAAGAAATTATAACGAGTGGTGATAATCTTGATAAAGAG 660

Alt CCAGAGAAACAAAACGTTATTCCAGAAATTATAACGAGTGGTGATAATCTTGATAAAGAG 660

********************** *************************************

Ref GAGGTGAAGAATGACAACAAGAAGTCTGGACTCTCATCGGCCAGGTTCTTGAACAGAGTA 720

Alt GAGGTGAAGAATGACAACAAGAAGCCTGGATTCTCATCGGCCAGGTTCTTGAACAGAGTA 720

************************ ***** *****************************

Ref GCTAATAGGTTCGGTAAGAGAATCAATCAAAGTGTTTTGTCTGAGATTATCGGAAGTGGT 780

Alt GCTAATAGGTTCGGTAAGAGAATCAATCAAAGTGTTTTGTCTGAGATTATCGGAAGTGGT 780

************************************************************

Ref GGCCCACTTACTAGTACCACTACAAGTCACACAACTACTACTACAAGTGTCACCATTAAC 840

Alt GGCCCACTTACTAGTACCACTACAAGTCACACAACTACTACTACAAGTGTCACCATTAAC 840

************************************************************

Ref TCCGAATCAGATAAGTCAATTAGCTCTTCCTTCACACCAACCTCATCAGATCTTCTATGC 900

Alt TCCGAATCAGATAAGTCAATTAGCTCTTCCTTCACACCAACCTCATCAGATCTTCTATGC 900

************************************************************

Ref CAGATGACCGTTAACGGTAACGCTACATCGTCTCCGTCCACATTCTCTGATGCATCCGTT 960

Alt CAGATGACAGTTAACGGTAACGCTACATCGTCTCCGTCCACATTCTCTGATGCATCCGTT 960

******** ***************************************************

Ref AATGATAGTTTAATGTACTGTGATAATGAGGATAATCTCGGATTCTCAAATTTTCTGAAT 1020

Alt AATGATAGTTTAATGTACTGTGATAATGAGGATAGTCTCGGATTCTCAAATTTTCTGAAT 1020

********************************** *************************

Ref GATGAAGATTTCATGATGTTCGGAGAGTCTTGTGTTGACAACACTGAGTTCATGAAAGAA 1080

Alt GATGAAGATTTCATGATGTTCGGAGAGTCTTGTGTTGACAACACTGAGTTCATGAAAGAA 1080

************************************************************

Ref CTTACGAGCTTTCTTCAGGAGGATGTGAGTGACGACGTCCAGGTGATGCCCGTCAATGAA 1140

Alt CTTACGAGCTTTCTTCAGGAGGATGTGAGTGACGACGTCCAGGTGATGCCCGTCAATGAA 1140

************************************************************

Ref CATAAAGACAATATTGAAGAGACTGATAACTATTTTGCA 1179

Alt CATAAAGACAATATTGAAGAGACTGATAACTATTTTGCA 1179

***************************************

**Protein alignment**

Ref MFELSNQKIMCSIVMIICPLSHFVSTLSFSIFFFSLFHISLGNNRFERDQNNHKNQELKM 60

Alt MFELSNQKIMCSIVMIICPLSHFVSTLSFSIFFFSLFHISLGNNRFERDQNNHKNQELKM 60

************************************************************

Ref VRTPCCKAELGLKKGAWTPEEDQKLVSYVNRHGEGGWRTLPEKAGLKRCGKSCRLRWANY 120

Alt VRTPCCKAELGLKKGAWTPEEDQKLVSYVNRHGEGGWRTLPEKAGLKRCGKSCRLRWANY 120

************************************************************

Ref LRPDIKRGEFTEDEERSIISLHALHGNKWAAIARGLPGRTDNEIKNHWNTHIKKLLIKKG 180

Alt LRPDIKRGEFTEDEERSIISLHALHGNKWAAIARGLPGRTDNEIKNHWNTHIKKLLIKKG 180

************************************************************

Ref VDPVTHKSLISDKSENLPEIPEKQNVIQEIITSGDNLDKEEVKNDNKKSGLSSARF**LN**RV 240

Alt VDPVTHKSLISDKSENLPEIPEKQNVIPEIITSGDNLDKEEVKNDNKKPGFSSARF**LN**RV 240

*************************** ******************** *:*********

Ref **A**NRFGKRINQSVLSEIIGSGGPLTSTTTSHTTTTTSVTINSESDKSISSSFTPTSSDLLC 300

Alt **A**NRFGKRINQSVLSEIIGSGGPLTSTTTSHTTTTTSVTINSESDKSISSSFTPTSSDLLC 300

************************************************************

Ref QMTVNGNATSSPSTFSDASVNDSLMYCDNEDNLGFSNFLNDEDFMMFGESCVDNTEFMKE 360

Alt QMTVNGNATSSPSTFSDASVNDSLMYCDNEDSLGFSNFLNDEDFMMFGESCVDNTEFMKE 360

*******************************.****************************

Ref LTSFLQEDVSDDVQVMPVNEHKDNIEETDNYFA 393

Alt LTSFLQEDVSDDVQVMPVNEHKDNIEETDNYFA 393

*********************************

Residue 208 – glutamine to proline

Residue 229 – serine to proline

Residue 231 – leucine to phenylalanine

Residue 332 – asparagine to serine

Functional domains based on Arabidopsis sequence

**MYC-interaction motif (MIM)**

**JAZ5 (aka TIFY 11A)**

**DNA alignment**

Ref ATGTCGAGAAATGAAGATGGTAAGGCACAACCGCCGGAGAAGTTTAACTTTACCCGGAGA 60

Alt ATGTCGAGAAATGAAGATGGTAAGGCACAACCGCCGGAGAAGTTTAACTTTACCCGGAGA 60

************************************************************

Ref TGTAGTTTGCTCAGCCGTTACTTGAAGGAGAAGGGTAGTTTCGGGAATATAGATCTTGGA 120

Alt TGTAGTTTGCTCAGCCGTTACTTGAAGGAGAAGGGTAGTTTCGGGAATATAGATCTTGGA 120

************************************************************

Ref TTAGTCCGAAAGCCCGAGTTGGATCTTGGGTTACCAGGAAACTATGATCAACAAGAGAAA 180

Alt TTAGTCCGAAAGTCCGAGTTGGATCTTGGGTTACCAGGAAACTATGATCAACAAGAGAAA 180

************ ***********************************************

Ref CAAAATGTGATGCATAAGGCAAAAGGCGAACTCTCTAGCTCATCTGGAGGCAAAGCCAAA 240

Alt CAAAATGTGATGCATAAGGCAAAAGGCGAACTCTCTAGCTCATCTGGAGGCAAAGCCAAA 240

************************************************************

Ref GCTACCAATCTCAGTGAACACCCAGATGCAGCAAGTTCACAGCTGACAATATTCTTTGGA 300

Alt GCTACCAATCTCAGTGAACACCCAGATGCAGCAAGTTCACAGCTGACAATATTCTTTGGA 300

************************************************************

Ref GGAAAAGTTTTAGTATACAATGAGTTTCCTGCAGAGAAAGCTAAAGAGATAATACAAGTA 360

Alt GGAAAAGTTTTAGTATACAATGAGTTTCCTGCAGAGAAAGCTAAAGAGATAATACAAGTA 360

************************************************************

Ref GCAAAAGAAGCCAAGCCTGAGACTGAGATCAACACTCAGACACAAATCAATGACCACAAG 420

Alt GCAAAAGAAGCCAAGCCTGTGACTGAGATCAACACTCAGACACAAATCAATGACCACAAG 420

******************* ****************************************

Ref AACAAAAGCAACATGGTTCTTCCGGATCTCAACGAGCCCACAGACTTTGCTGATGTCAAT 480

Alt AACAAAAGCAACATGGTTCTTCCGGATCTCAACGAGCCCACAGACTTTGCTGATGTCAAT 480

************************************************************

Ref CAGCAACAACAACAACAACAAAACCAGCTCGTGGAACGTATAGCACGTAGAGCTTCCCTT 540

Alt CAACAACAACAACAACAACAAAACCAGCTCGTGGAACGTATAGCACGTAGAGCTTCCCTT 540

** *********************************************************

Ref CATCGGTTCTTTGCTAAACGTAAAGACAGAGCTGTGGCTAGAGCTCCATACCAAGTTAAC 600

Alt CATCGGTTCTTTGCTAAACGTAAAGACAGAGCTGTGGCTAGAGCTCCATACCAAGTTAAC 600

************************************************************

Ref CAAAACGCTGGTCGTCATCATTATCCTCCCAAGCCAGAGACTCTGCCCGGTCAGCAGCGA 660

Alt CAAAACGCTGGTCGTCATCATTATCCTCCCAAGCCAGAGACTCTGCCCGGTCAGCAGCGA 660

************************************************************

Ref GAGCAGGGACAGTCGTCACAACGACCGGACACTGCTGTTGCTCAAACCGTTTCCCATCCC 720

Alt GAGCAGGGACAGTCGTCACAACGACCGGACACTGCTGTTGCTCAAACCGTTTCCCATCCC 720

************************************************************

Ref AAACCAGAATGTGCTAAAGATATGTTGATGGAAGTTAAGGGAGAGGGCCAATGTTCGAAA 780

Alt AAACCAGAATGTGCTAAAGATATGTTGATGGAAGTTAAGGGAGAGGGCCAATGTTCGAAA 780

************************************************************

Ref GATCTCGAACTTAGGCTA 798

Alt GATCTCGAACTTAGGCTA 798

******************

**Protein alignment**

Ref MSRNEDGKAQPPEKFNFTRRCSLLSRYLKEKGSFGNIDLGLVRKPELDLGLPGNYDQQEK 60

Alt MSRNEDGKAQPPEKFNFTRRCSLLSRYLKEKGSFGNIDLGLVRKSELDLGLPGNYDQQEK 60

******************************************** ***************

Ref QNVMHKAKGELSSSSGGKAKATNLSEHPDAASSQL**TIFFGG**KVLVYNEFPAEKAKEIIQV 120

Alt QNVMHKAKGELSSSSGGKAKATNLSEHPDAASSQL**TIFFGG**KVLVYNEFPAEKAKEIIQV 120

************************************************************

Ref AKEAKPETEINTQTQINDHKNKSNMVLPDLNEPTDFADVNQQQQQQQNQLVERIARRASL 180

Alt AKEAKPVTEINTQTQINDHKNKSNMVLPDLNEPTDFADVNQQQQQQQNQLVERIARRASL 180

****** *****************************************************

Ref HRFFAKRKDRAVARAPYQVNQNAGRHHYPPKPETLPGQQREQGQSSQRPDTAVAQTVSHP 240

Alt HRFFAKRKDRAVARAPYQVNQNAGRHHYPPKPETLPGQQREQGQSSQRPDTAVAQTVSHP 240

************************************************************

Ref KPECAKDMLMEVKGEGQCSKDLELRL 266

Alt KPECAKDMLMEVKGEGQCSKDLELRL 266

**************************

Residue 45 – proline to serine

Residue 127 – glutamic acid to valine

Functional domains based on Arabidopsis sequence

**TIFY binding domain**
