# Supplementary figures and images for: Quantitative trait loci analysis of glucosinolate, sugar, and organic acid concentrations in Eruca vesicaria subsp. sativa
Source: Mol Hortic. 2022 Oct 10;2:23. doi: 10.1186/s43897-022-00044-x (PMC10515263; doi:10.1186/s43897-022-00044-x)

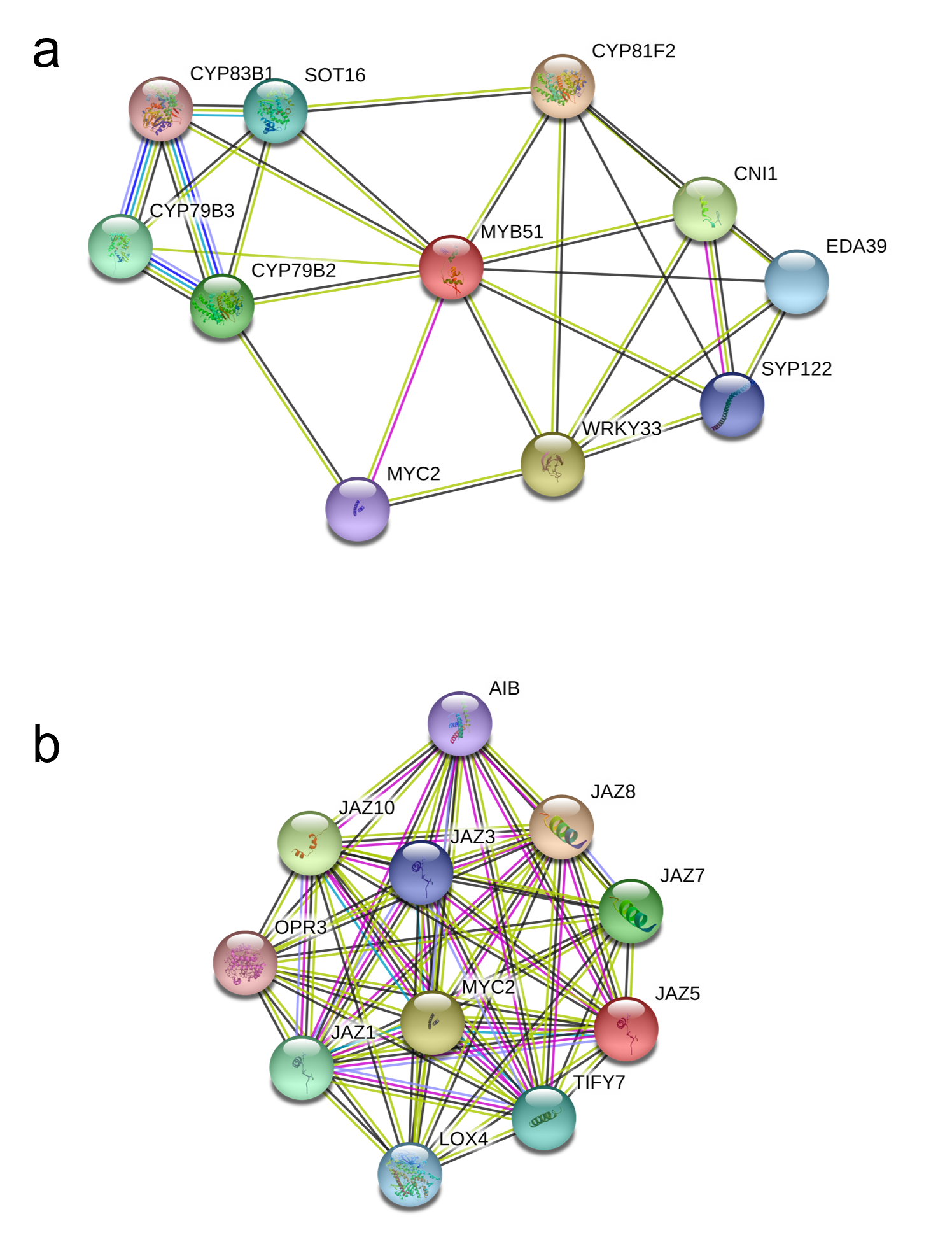

Supplement: Supplementary file 9 — Additional file 9. Protein-protein-interaction networks of Arabidopsis thaliana highlighting known links between MYB51 and MYC2 (a), and JAZ5 (also known as TIFY 11A) and MYC2. [file 43897_2022_44_MOESM9_ESM.tif]
